# Stat3 activation-triggered transcriptional networks govern the early stage of HBV-induced hepatic inflammation

**DOI:** 10.1128/mbio.03068-23

**Published:** 2024-03-05

**Authors:** Jinglin Tang, Jiaxuan Zhang, Gaoli Zhang, Wenhui Peng, Ning Ling, Yingzhi Zhou, Hongmei Xu, Hong Ren, Min Chen

**Affiliations:** 1 Department of Infectious Diseases, Key Laboratory of Molecular Biology for Infectious Diseases (Ministry of Education), Institute for Viral Hepatitis, Second Affiliated Hospital of Chongqing Medical University, Chongqing, China; 2 Department of Transfusion Medicine, West China Hospital of Sichuan University, Chengdu, China; 3 Department of Laboratory Medicine, Bishan Hospital of Chongqing Medical University, Bishan Hospital of Chongqing, Chongqing, China; 4 Department of Infection, Ministry of Education Key Laboratory of Child Development and Disorders, National Clinical Research Center for Child Health and Disorders, Children's Hospital of Chongqing Medical University, Chongqing, China; Catholic University of America, Washington, DC, USA; Memorial Sloan Kettering Cancer Center, New York, New York, USA

**Keywords:** chronic hepatitis B, chronic HBV carriers, liver inflammation, Stat3, immune responses

## Abstract

The chronic carrier state of the hepatitis B virus (HBV) often leads to the development of liver inflammation as carriers age. However, the exact mechanisms that trigger this hepatic inflammation remain poorly defined. We analyzed the sequential processes during the onset of liver inflammation based on time-course transcriptome and transcriptional regulatory networks in an HBV transgenic (HBV-Tg) mice model and chronic HBV-infected (CHB) patients (data from GSE83148). The key transcriptional factor (TF) responsible for hepatic inflammation occurrence was identified and then validated both in HBV-Tg mice and liver specimens from young CHB patients. By time-course analysis, an early stage of hepatic inflammation was demonstrated in 3-month-old HBV-Tg mice: a marked upregulation of genes related to inflammation (Saa1/2, S100a8/9/11, or Il1β), innate immunity (Tlr2, Tlr7, or Tlr8), and cells chemotaxis (Ccr2, Cxcl1, Cxcl13, or Cxcl14). Within CHB samples, a unique early stage of inflammation activation was discriminated from immune tolerance and immune activation groups based on distinct gene expression patterns. Enhanced activation of TF Stat3 was strongly associated with increased inflammatory gene expression in this early stage of inflammation. Expression of phosphorylated Stat3 was higher in liver specimens from young CHB patients with relatively higher alanine aminotransferase levels. Specific inhibition of Stat3 activation significantly attenuated the degree of liver inflammation, the expression of inflammation-related genes, and the inflammatory monocytes and macrophages in 3-month-old HBV-Tg mice. Stat3 activation is essential for hepatic inflammation occurrence and is a novel indicator of early-stage immune activation in chronic HBV carriers.

**IMPORTANCE:**

Until now, it remains a mystery that chronic hepatitis B virus (HBV)-infected patients in the “immune tolerance phase” will transition to the “immune activation phase” as they age. In this study, we reveal that Stat3 activation-triggered hepatic transcriptional alterations are distinctive characteristics of the early stage of immune/inflammation activation in chronic HBV infection. For the first time, we discover a mechanism that might trigger the transition from immune tolerance to immune activation in chronic HBV carriers.

## INTRODUCTION

Globally, an estimated 296 million people had chronic hepatitis B virus (HBV) infection in 2019, two-thirds of whom resided in the Western Pacific region. Additionally, approximately 880,000 people die each year from hepatitis B and related complications, such as liver cirrhosis and hepatocellular carcinoma (1).

Infants infected with HBV are more susceptible to developing chronic HBV infection (CHB). Typically, these chronic infections first undergo an early phase called the “immune-tolerant (IT) phase” or “chronic HBV carrier” phase. The IT phase is characterized by high levels of serum HBV DNA, the presence of HBeAg, normal serum alanine aminotransferase (ALT) levels, and minimal or no liver necroinflammation or fibrosis on biopsy (2, 3). After the age of 20–25 years, the IT phase might gradually transition to the immune clearance or immune-active phase (IA) with declining levels of HBV DNA, elevated ALT levels, and amplified hepatic inflammation. However, the exact mechanisms of this transition are still largely unknown.

The occurrence of hepatitis in the IA phase was traditionally considered the result of HBV-specific immune activation during the process of chronic HBV infection. However, this concept has recently been challenged. Studies have shown that IT HBV-infected adolescents did not display the characteristics of tolerogenic T cells. On the contrary, HBV-specific T cells from IT patients were superior in quantity and function compared to IA patients (4). Therefore, the HBV-specific immune response might not be the direct driving factor associated with the conversion from the IT to IA phase. Some researchers have hypothesized that a higher propensity of inflammation response or certain pro-inflammatory reactions in adult CHB patients might directly induce liver inflammatory events during the “immune-active phase” (5, 6). Collecting liver tissue longitudinally in patients makes it very challenging to monitor and investigate the process of liver immune-inflammation activation in these chronic HBV carriers.

In the latest studies, researchers aimed to identify the factors related to “immune activation” by comparing the transcriptomic profiles of liver biopsies or hepatic immune cells from CHB patients in different clinical phases (7). They observed enhancement of hepatic immune-related gene expression or myeloid cell infiltration in both IT and IA CHB patients, with more significant differences in IA patients when compared to healthy controls (HCs). However, the differences in age and disease progression in these patients made it difficult to further explore the characteristics of the early phase of immune activation or the transition from the immune tolerance phase to the immune activation phase.

Currently, there is a lack of small animal models that reproduce human-like HBV infections. But HBV replication can be achieved in non-susceptible species (such as mouse) when HBV genome is introduced into hepatocytes. HBV transgenic mice (HBV-Tg) with high levels of HBV viral load or HBs/HBx antigen have been reported to spontaneously develop hepatic injury with age and utilized for studying HBV-related hepatic fibrosis and hepatocarcinoma (8–10). In this study, we explored the mechanisms contributing to the occurrence of immune-active phase by observing the dynamics of hepatic gene expression profiles in HBV-Tg mice at 1, 3, and 6 months. According to reported articles, mice aged 1, 3, or 6 months corresponded approximately to human aged 0.5, 21, or 30 years (11). As we know, the majority of neonates exposed to HBV develop chronic infection and enter a prolonged immune-tolerant phase, followed by immune-active phase (median age of onset is 30 years) (12). Therefore, dynamic observation in these HBV-Tg mice might reflect the progression from immune tolerance to immuno-inflammatory activation in chronic HBV carriers. Overall, this study provides new insights into the mechanisms of inflammation development in chronic HBV carriers.

## MATERIALS AND METHODS

### Mice

HBV transgenic mice (male) with C57BL/6N background carrying a 1.28-fold overlength HBV genome (genotype A) were purchased from Vitalstar Biotechnology (Beijing, China). Wild-type (WT) C57BL/6N mice (male) were used as controls. HBV-Tg mice and WT controls were sacrificed at three time points: 1, 3, and 6 months of age. Five to seven mice were examined for each age group. All mice were housed under the sterile conditions, controlled temperature, and light conditions (22°C–25°C and 12 h:12 h light/dark cycle), with *ad libitum* access to water and diet. All mouse handling and experiments were approved by the Ethics Committee of Chongqing Medical University.

### Detection of HBV-DNA, HBsAg, HBeAg, ALT, and AST

Blood samples were collected from HBV-Tg and WT mice at indicated age. Plasma was separated and stored frozen for subsequent batch analysis. According to the manufacturer’s instructions, quantification of HBV DNA was performed by real-time quantitative PCR (qPCR) with the COBAS AmpliPrep/COBAS TaqMan HBV Test Kit (v2.0) (Roche Diagnostic Systems, NJ, USA). Levels of HBsAg and HBeAg were detected by chemiluminescence microparticle immunoassay with Alinity HBsAg and HBeAg Detection Kits (Abbott Laboratories, IL, USA). ALT and AST levels were measured by the Hitachi 7600 Series automatic biochemical analyzer (Hitachi, Tokyo, Japan).

### Histology and immunohistochemistry staining

Liver tissues from HBV-Tg and WT mice at different time points were fixed in 4% paraformaldehyde for 24 h at room temperature (RT), dehydrated using an ethanol gradient (70%, 80%, 90%, and 100%), embedded in paraffn, and subsequently sectioned at 4 um thickness. Paraffin slides were deparaffinized and then rehydrated by a series of graded alcohols until water is used. Then, these slides were stained using hematoxylin-eosin (H&E) for 10 minutes at RT for histological examination. For immunohistochemistry staining, the dehydrated sections were incubated with 3% hydrogen peroxide solution for 15 minutes at room temperature, treated with 0.01 M sodium citrate buffer for heat-induced antigen retrieval, and then blocked with 10% normal goat serum. Next, sections were incubated with primary antibodies diluted in blocking buffer overnight at 4°C. Monoclonal mouse anti-HBcAg (cloning number: MX104) were purchased from MXB Biotechnologies (MXB Biotechnologies, Fuzhou, China) and monoclonal rabbit anti-Stat3 (phospho Y705) (cloning number: EP2147Y) from Abcam (Cambridge, UK). Ready-to-use biotinylated goat anti-rabbit, mouse IgG secondary antibody was used to conjugate the primary antibody, and finally, protein was visualized by avidin-labeled horseradish peroxidase and diaminobenzidine staining (MXB Biotechnologies, Fuzhou, China). Cells positive for HBcAg or Stat3 were counted on the whole section. Data were expressed as median number per square millimeter.

### RNA sequencing and raw data processing

Liver tissues were collected from HBV-Tg and WT mice at indicated age and frozen immediately in liquid nitrogen. Total RNA was extracted using TRIzol reagent (Life technologies) following the manufacturer’s instructions. The RNA integrity was assessed by Agilent 2100 Bioanalyzer. Only samples producing a 28S/18S ratio higher than 1.1 or an RNA integrity number higher than 7.5 were used for further RNA sequencing.

RNA sequencing and raw data processing were all performed at Beijing Genomics Institute (Shenzhen, China). In brief, sequencing libraries were generated by mRNA isolation, mRNA fragmentation, cDNA synthesis, and PCR amplification. After library quality control, sequencing was performed using DNBSEQ platform. The sequencing data were filtered with SOAPnuke; afterward, clean reads were obtained and stored in FASTQ format. Bowtie2 was applied to align the clean reads to the mouse reference genome (GRCm38.p6) (13), and expression level of gene was calculated by RSEM (v1.3.1).

### Hepatic gene expression data sets of CHB patients from GEO database

Microarray transcriptome data of liver biopsy together with corresponding clinical information from CHB patients and HCs were obtained from the Gene Expression Omnibus (GEO) database (GSE83148). Among these CHB patients, 13 patients had normal ALT (≤40 IU/mL) and AST levels (≤35 IU/mL) and high HBV DNA load (>10^6^ copies/mL), and considered in immune tolerance phase. And 19 patients had abnormal ALT (>40 IU/mL) and AST levels (>35 IU/mL), and relatively lower HBV DNA load (<10^6^ copies/mL), considered in immune-active phase. Finally, the gene expression matrix of 32 CHB patients and 6 HCs was obtained.

### Bioinformatics analysis of gene expression profiles

For RNA-seq data (read counts) and microarray data (normalized data), the subsequent bioinformatics analysis was performed in R software (v4.2.1).

- The differentially expressed genes (DEGs) were identified using the DESeq2 or limma package (14, 15). DEGs with *P* < 0.05 and |fold change (FC)| ≥2 were considered statistically significant.
- From the AmiGO 2 (http://amigo.geneontology.org/amigo/landing) database, 2,274 immune/inflammation-related genes (IRGs) for human or 2,089 IRGs for mouse were obtained (Table S1).
- The ConsensusClusterPlus package was used for consistent clustering to determine the subgroup of CHB patients from GSE83148 (16).
- Weighted gene co-expression network analysis (WGCNA) was used to screen for co-expressed gene modules that were markedly correlated with different groups or traits (17).
- Functional gene set enrichment analysis (GSEA) was conducted using R clusterProfiler package (18). Hallmark gene sets were collected from Molecular Signatures Database (https://www.gsea-msigdb.org/gsea/msigdb/mouse/genesets.jsp?collection=MH).
- Top 500 upregulated genes in each HBV-Tg group (compared to corresponding WT group) or CHB patient group (compared to HC) were submitted to the Metascape (http://metascape.org/gp/index.html) for Gene Ontology (GO) enrichment analysis (19). The results of enrichment analysis were used to build biological networks and further visualized with Cytoscape (20).
- Transcription factor (TF) enrichment analysis was implemented by ChEA3 (https://maayanlab.cloud/chea3/), ENCODE (https://www.encodeproject.org/), or TRRUST (https://www.grnpedia.org/trrust/) database.
- The fraction of immune cells infiltration was estimated using CIBERSORT algorithm (21). The feature matrix of 25 different leukocyte subpopulations in mice (srep40508-s1) was referenced from Chen et al. (22).

### Quantification of gene expression by real-time PCR

Liver homogenates were prepared by grinding tissue in liquid nitrogen and then by bead-based homogenization in 500 µL TRIzol reagent (Life Technologies). Total RNA was extracted with RNA Isolation Kit (R2052, ZYMO Research Co., Irvine, CA, USA), according to the manufacturer’s instructions. First-strand cDNA was generated from about 1 µg of total RNA by PrimeScriptTM RT Reagent Kit with gDNA Eraser (RR047A, Takara Co., Japan). Real-time PCR was performed with TB Green Premix Ex Taq II Kit (RR820A, TaKaRa, Japan). The relative level of gene expression was calculated by the comparative cycle threshold (Ct) method (2^−ΔΔCt^). The housekeeping gene GAPDH was used to normalize the original Ct values in our current experiments. All samples were tested in triplicate. The primers used were listed in Table S2.

### Western blot analysis

Total proteins were extracted from the liver tissues using the Whole-Protein Extraction Kit (KGP250, KeyGEN BioTECH, China). The protein concentration was detected using a Bicinchoninic Acid Kit (KGPBCA, KeyGEN BioTECH, China). Protein samples were diluted into 5× SDS loading buffer and denatured by boiling at 100°C for 5 minutes. Protein samples were electrophoresed on 10% sodium dodecyl sulfate polyacrylamide gels and transferred to PVDF membranes. Membranes were blocked with 5% non-fat milk in TBST buffer for 1 h at room temperature and then incubated overnight at 4°C with the following primary antibody: mouse anti-Stat3 (9D8, Abcam), rabbit anti-Stat3 (phospho Y705) (EP2147Y, Abcam), rabbit anti-β-actin (13E5, CST), rabbit anti-NF-kB p65 (D14E12, CST), or rabbit anti-NF-kB p65 (Phospho Ser536) (93H1, CST). Then, membrane was incubated with the secondary antibody (7074P2, 7076P2, CST) for 2 h at ambient temperature. Finally, protein bands were detected by enhanced chemiluminescence and gel imaging system (BioRad). Quantification of protein expression was performed by ImageJ.

### Intrahepatic and peripheral blood leukocytes isolation

Intrahepatic leukocytes were separated as described previously (23, 24). Briefly, the liver tissues were grinded on a 200-gauge metal mesh in PBS. All cell suspension was collected and centrifuged at 50 × *g* for 5 minutes. The supernatant was separated and centrifuged at 2,000 × rpm for 10 minutes. Then the cell pellet was resuspended in 40% Percoll (Cytiva) and centrifuged at 860 × *g* for 15 minutes. Red blood cells (RBCs) were lysed with 0.8% ammonium chloride (NH_4_Cl) solution and washed twice with PBS. Peripheral blood leukocytes were isolated by RBCs lysis with NH_4_Cl solution and washed twice with PBS. Leukocytes were harvested for flow cytometric analysis.

### Flow cytometry

Leukocyte suspension was pre-incubated with anti-CD16/CD32 for 5 minutes for blocking Fc receptors and stained with the specific fluorescent-labeled monoclonal antibodies (Abs) at 4°C for 45 minutes in darkness. The following monoclonal Abs were used in this study: PE/Cyanine7 anti-mouse CD45 (S18009F, BioLegend), Brilliant Violet 650 anti-mouse/human CD11b (M1/70, BioLegend), PerCP/Cyanine5.5 anti-mouse Ly-6C (HK1.4, BioLegend), and Brilliant Violet 421 anti-mouse F4/80 (BM8, BioLegend). Cell samples were analyzed using a flow cytometer (CytoFLEX, Beckman Coulter, USA).

### Paraffin-embedded liver tissues of chronic HBV infection cases for p-Stat3 staining

To observe hepatic p-Stat3 expression in early stage of chronic HBV infection, a retrospective institutional archival search for liver biopsies of chronic HBV infection cases from July 2021 to December 2022 in Children’s Hospital of Chongqing Medical University was performed. Clinical and laboratory data, and sections with H&E stain, HBsAg, and HBcAg stain were reviewed. The inclusion criteria of cases were as follows: 3–18 years old, seropositivity for HBsAg and HBeAg, high HBV DNA level (>1 × 10^6^ IU/mL), normal or slightly elevated serum ALT levels (≤2× ULN). Exclusion criteria were antiviral therapy or immunosuppressive therapy, coinfection with other virus, or significant liver disease. Finally, nine HBV-infected liver tissues (formalin fixed and paraffin embedded) were obtained from the Department of Pathology. This protocol was conducted according to the guidelines of the declaration of Helsinki and approved by the Ethics Committee of the Children’s Hospital of Chongqing Medical University.

### Inhibition of Stat3 activation *in vivo*

Stattic, a small molecule inhibitor of Stat3 activation, was used in this study. Two-month-old HBV-Tg and WT mice were divided into three groups (*n* = 5): the WT/Vehicle group, the HBV-tg/Vehicle group, and the HBV-tg/Stattic group. Stattic (S7024) was purchased from Selleck. The mice in the Stattic group were injected intraperitoneally with Stattic solution (5 mg/kg, dissolved in DMSO:PEG300:Tween80:saline =  1:8:1:10) once every 3 days for 4 weeks. The mice in the Vehicle group were only treated with the same volume of solvent.

### Statistical analysis

Each experimental group included at least five biological replicates. Data were expressed as mean ± SEM for continuous variables and category frequency for categorical variables. Wilcoxon rank-sum test was used for comparing the means of two independent samples, Kruskal-Wallis test with Dunn’s correction or one-way ANOVA with Sidak’s correction was used for multiple comparisons of three or more group means, and Pearson chi-square test was used for category frequencies. All statistical tests were two tailed, and statistical significance was set at *P* < 0.05. Normalization was used for the analysis of gene or protein expression data, obtained by RNA-seq, gene chip, qPCR, or western blot method. ComplexHeatmap (25) and ggplot2 were used to plot heat maps and other graphs. All statistical analyses were performed using GraphPad Prism (v 8.0) and R (v 4.2.1).

## RESULTS

### A study of age-related hepatic immune/inflammatory activation through longitudinal analysis of hepatic histology and transcriptome in an HBV-Tg mouse model

**Fig 1:**
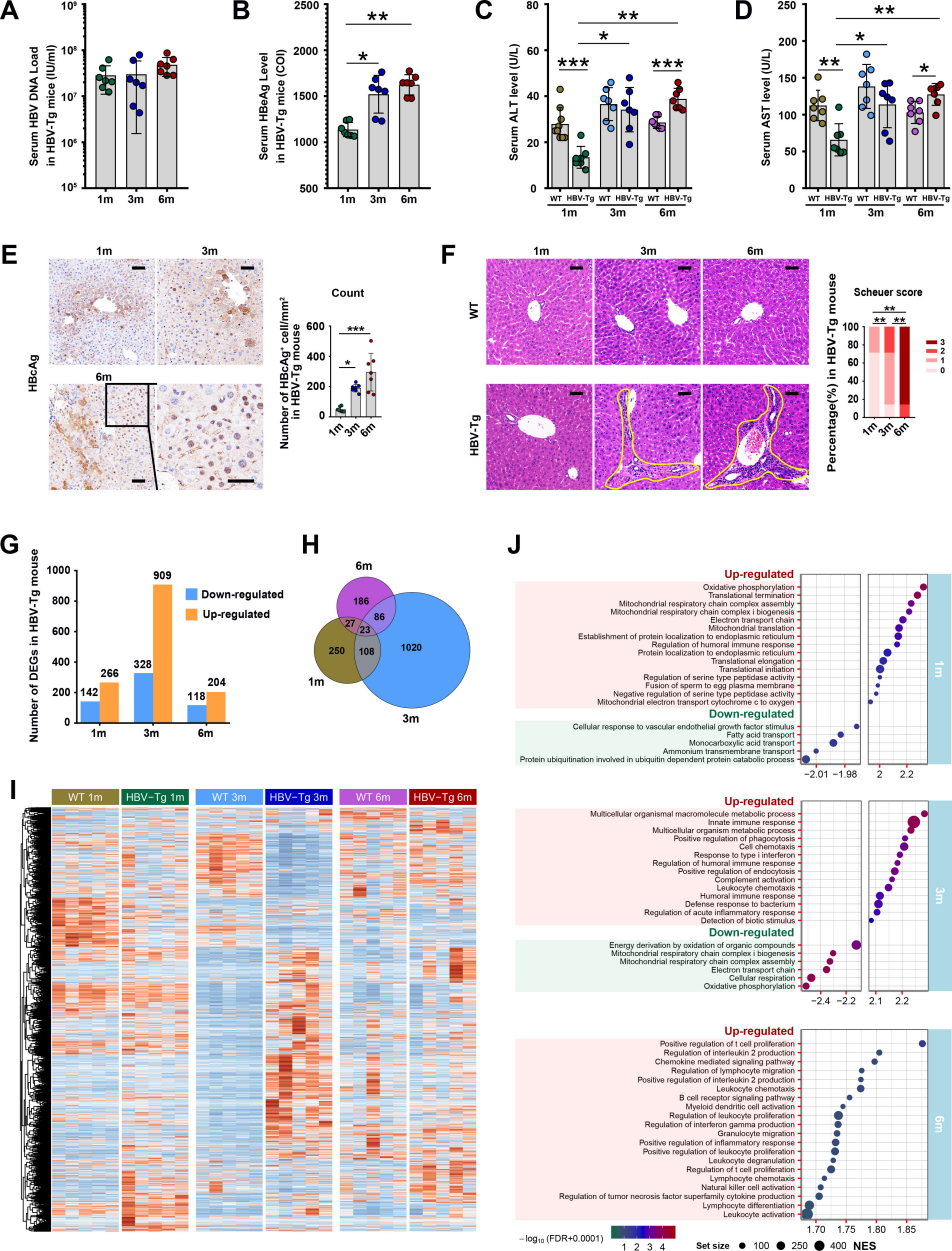
Longitudinal analysis for hepatic histology and transcriptome in an HBV-Tg mouse model. The 1-, 3-, and 6-month-old HBV-Tg mice and their WT controls (C57BL/6N) at the same age were used in this study (*n* = 7/group). Blood HBV DNA load and HBeAg level were tested in HBV-Tg mice (**A and B**). Serum ALT and AST levels were examined in both HBV-Tg and WT mice (**C and D**). (**E**) Representative images of HBcAg expression detected by immunohistochemical staining on liver sections from HBV-Tg mice and quantification of HBcAg-positive hepatocytes (brown) per square millimeter. Scale bar: 50 μm. (**F**) Representative images of H&E staining of liver tissue sections from WT mice (top row) and HBV-Tg mice (bottom row), and the inflammation grading in liver sections from HBV-Tg mice evaluated with Scheuer scoring systems. Irregular golden curves marked typical portal area inflammation. (**G**) The number of up- and downregulated DEGs in 1-, 3-, or 6-month-old HBV-Tg mice compared to corresponding WT mice (*n* = 5/group). (**H**) Venn diagram of overlapping number of DEGs among HBV-Tg mice group. (**I**) Heat map of all DEGs in both or either of 1-, 3-, or 6-month-old HBV-Tg mice group. Gene expression values were normalized by row (scale). (**J**) The top 20 significantly enriched GO terms in 1-, 3-, or 6-month-old HBV-Tg mice compared to corresponding WT mice by GSEA analysis. Gene sets were GO biological processes and corrected for *P* values using FDR. (**A–E**) Each dot represented an individual mouse. Each bar summarized a data set using the mean and standard error of the data set. *P < 0.05, **P < 0.01, ***P < 0.001. 1m, 1 month old; 3m, 3 months old; 6 m, 6 months old; Schuer score, an inflammation-based score evaluated by Scheuer scoring systems; NES, normalized enrichment score; FDR, false discovery rate.

To investigate the dynamic inflammatory process in the context of high HBV viral load, we observed the HBV-Tg mouse model continuously at 1, 3, and 6 months. HBV-Tg mice exhibited consistently high levels of serum HBV DNA (greater than 1 × 10^7^ IU/mL) and HBeAg (greater than 500 IU/mL) from 1 to 6 months (Fig. 1A and B). There was a significant increase in serum HBeAg levels at 3 and 6 months compared to 1 month (*P* < 0.05 and *P* < 0.01, respectively) (Fig. 1B). Serum ALT or AST levels gradually increased with age among HBV-Tg mice, and the 3- and 6-month groups displayed significantly higher levels compared to the 1-month group (*P* < 0.05 and *P* < 0.01, respectively). Interestingly, when compared to age-matched wild-type mice, HBV-Tg mice exhibited significantly higher ALT or AST values at 6 months (*P* < 0.001 and *P* < 0.05, respectively), whereas lower values were noted at 1 month (*P* < 0.001 and *P* < 0.01, respectively) (Fig. 1C and D). HBcAg expression was observed in both the hepatocyte nucleus and cytoplasm. The 3- and 6-month-old HBV-Tg mice displayed a higher number of HBcAg-positive hepatocytes compared to the 1-month group (*P* < 0.05 and *P* < 0.001, respectively) (Fig. 1E). Histological analysis revealed that HBV-Tg mice exhibited minimal or very few liver-infiltrating immune cells at 1 month (Scheuer score 0–1), which significantly increased in hepatic portal inflammation at 3 months (Scheuer score 0–2) (*P* < 0.01) and 6 months (Scheuer score 2–3) (*P* < 0.01) (Fig. 1F).

Subsequently, the dynamic hepatic gene expression profiles in these HBV-Tg mice were assessed by comparing the entire hepatic transcriptome of HBV-Tg mice at 1, 3, or 6 months with sex- and age-matched WT mice. In total, 408, 1,237, and 322 significantly differentially expressed genes were identified in 1, 3, or 6-month-old HBV-Tg mice (log2|FC| > 1, *P* < 0.05), of which 266 (65%), 909 (73%), and 204 (63%) were upregulated, respectively (Fig. 1G). A complete list of all DEGs can be found in Table S3. Additionally, 23 DEGs were common among all HBV-Tg groups, 131 were shared between the 1- and 3-month groups, 50 between the 1- and 6-month groups, and 109 DEGs between the 3- and 6-month groups (Fig. 1H). A heatmap of all 1,700 DEGs in either or both 1, 3, and 6 months revealed distinct transcriptional signatures for these three time points (Fig. 1I).

Gene Ontology biological process-based GSEA highlighted significant alterations in hepatic transcriptomes of HBV-Tg mice, enriched in 219, 503, or 77 pathways at 1, 3, or 6 months (|NES| >1, *P* < 0.05, and FDR <0.25), with 34 (15%), 409 (81%), or 77 (100%) upregulated, respectively (NES >1) (Table S4). The top 20 significantly enriched GO terms (ranked by |NES|) in 1-, 3-, or 6-month-old HBV-Tg mice are presented in Fig. 1J. Notably, at 1 month, the top 5 upregulated pathways were associated with mitochondrial function, including oxidative phosphorylation, mitochondrial respiratory chain complex assembly, electron transport chain, among others. Meanwhile, 3-month-old HBV-Tg mice displayed the most significantly upregulated pathways related to innate immune responses, type I interferon response, complement activation, inflammation response, and acute phase response. Finally, the 6-month-old HBV-Tg mice exhibited the highest enrichment scores in pathways linked to T cell proliferation and B cell activation (Fig. 1J).

### Sharp increase in hepatic immune/inflammation-related gene expression and monocyte infiltration in 3-month-old HBV-Tg mice

The aforementioned findings imply the possibility of age-dependent activation of hepatic immune/inflammation-related pathways in HBV-Tg mice. Subsequently, the investigation focused on the profiles of immune/inflammation-related genes (Table S1) and immune cells in these age-related processes.

**Fig 2:**
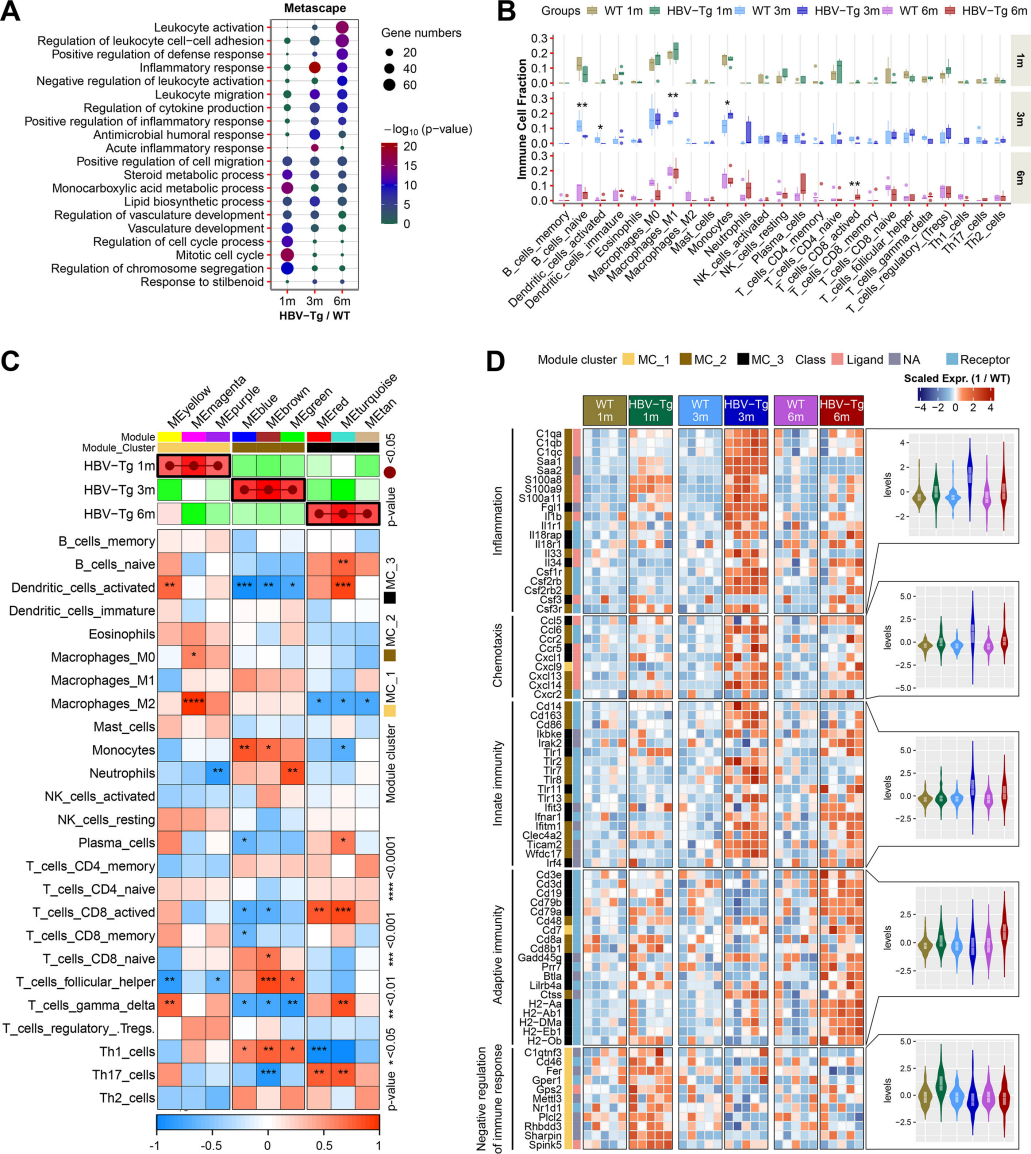
A sharp increase in hepatic inflammation-related genes expression and infiltration of monocytes and M1 macrophages in 3-month-old HBV-Tg mice. (**A**) Metascape enrichment analysis (GO-BP) for top 500 most upregulated genes in each HBV-Tg group (*n* = 5/group). (**B**) Intrahepatic infiltration of immune cells was analyzed by CIBERSORT. Only immune cell populations with significant differences in cell fraction between HBV-Tg mice and their WT controls were shown here. (**C**) Nine significant IRG modules were identified (from MEyellow to MEtan) by WGCNA. IRG modules positively correlated to 1-, 3-, or 6-month-old HBV-Tg mice were clustered into MC_1, MC_2, or MC_3. Heat map showed correlations between IRG modules and immune cell fractions analyzed by Pearson correlation analysis. (**D**) Heat map showed expression profiles of representative genes in the module cluster (MC_1, MC_2, or MC_3), including module markers, molecular classifications of encoded proteins, gene function annotation, and the overall expression level of these genes in different groups. Gene expression values were normalized by row (scale). *P < 0.05, **P < 0.01, ***P < 0.001. 1 m, 1 month old; 3 m, 3 months old; 6 m, 6 months old.

Within this context, the dynamics of upregulated pathways were further scrutinized using the top 500 most upregulated genes in each HBV-Tg group through Metascape GO-BP analysis. Pathways linked to leukocyte activation were progressively heightened, while inflammation responses exhibited a sharp increase at 3 months followed by a subsequent decline at 6 months. Notably, a significant surge in acute inflammatory response was specifically observed in the 3-month group (Fig. 2A).

Subsequently, the intrahepatic infiltration of immune cells was analyzed using CIBERSORT, which provided the proportions of 25 types of mouse immune cells (Fig. 2B). The results demonstrated that the fractions of hepatic monocytes and M1 macrophages were significantly increased in 3-month-old HBV-Tg mice (*P* = 0.032 and *P* = 0.0079, respectively), whereas naive B cells and activated dendritic cells exhibited marked reductions (*P* = 0.0079 and *P* = 0.025, respectively). Noteworthy changes in cell-type proportions were not detected at 1 and 6 months, except for an increased fraction of activated CD8^+^ T cells at 6 months (*P* = 0.0075) (Fig. 2B).

The exploration then turned to the expression patterns of immune/inflammation-related genes in HBV-Tg mice at different time points. Utilizing WGCNA, a total of nine significant IRG modules were identified (each represented by a different color), among which three exhibited positive correlations with specific time points (designated MC_1, MC_2, or MC_3) (Fig. 2C).

Subsequently, the relationships between these IRG modules and immune cell infiltration were examined. Particularly noteworthy was the positive correlation of distinct modules from the 1-, 3-, or 6-month age groups (MC_1, MC_2, or MC_3) with fractions of M2 macrophages at 1 month (*P* < 0.0001), monocytes and T helper cells at 3 months (*P* < 0.05), or activated CD8^+^ T cells and Th17 cells at 6 months, respectively (*P* < 0.01) (Fig. 2C).

Further delving into the functions of these IRG modules and their expression at different time points, a gene expression heatmap (Fig. 2D) depicted select representative IRGs within these modules, each associated with specific immune/inflammation-related biological processes. As anticipated, genes in the MC_1 module exhibited upregulation primarily in 1-month-old HBV-Tg mice, associated with negative regulation of inflammation and immunity. Conversely, genes related to inflammation and chemotaxis demonstrated remarkable upregulation in 3-month-old HBV-Tg mice, predominantly falling under the MC_2 module. Notably, the expression of innate immune-related genes was notably elevated at both 3 and 6 months, corresponding to the MC_2 and MC_3 modules, respectively. Furthermore, the upregulated adaptive immune-related genes were predominantly observed in the 6-month age group or the MC_3 module (Fig. 2D).

### Comparative analysis of hepatic IRGs signature between chronic HBV-infected patients and HBV-Tg mice

In this investigation, the hepatic expression patterns of immune/inflammation-related genes (Table S1) were compared across different age groups of HBV-Tg mice and various clinical stages of chronic HBV-infected patients. The gene expression chip data from GSE83148, containing uninfected controls and chronic HBV-infected patients with varying ALT, AST levels, and HBV DNA load, were obtained from the GEO database. Based on the natural course of chronic HBV infection, 13 patients were classified as being in the immune tolerance phase, characterized by normal ALT (≤40 U/L), AST (≤35 U/L) levels, and a high HBV-DNA load (>10^6^ copies/mL). On the other hand, 19 patients were categorized as being in the immune activation phase, exhibiting elevated ALT (>40 U/L) and AST (>35 U/L) levels, alongside a relatively low HBV-DNA load (<10^6^ copies/mL). Ultimately, data from 32 CHB patients and 6 uninfected controls (HC) were included and analyzed.

**Fig 3:**
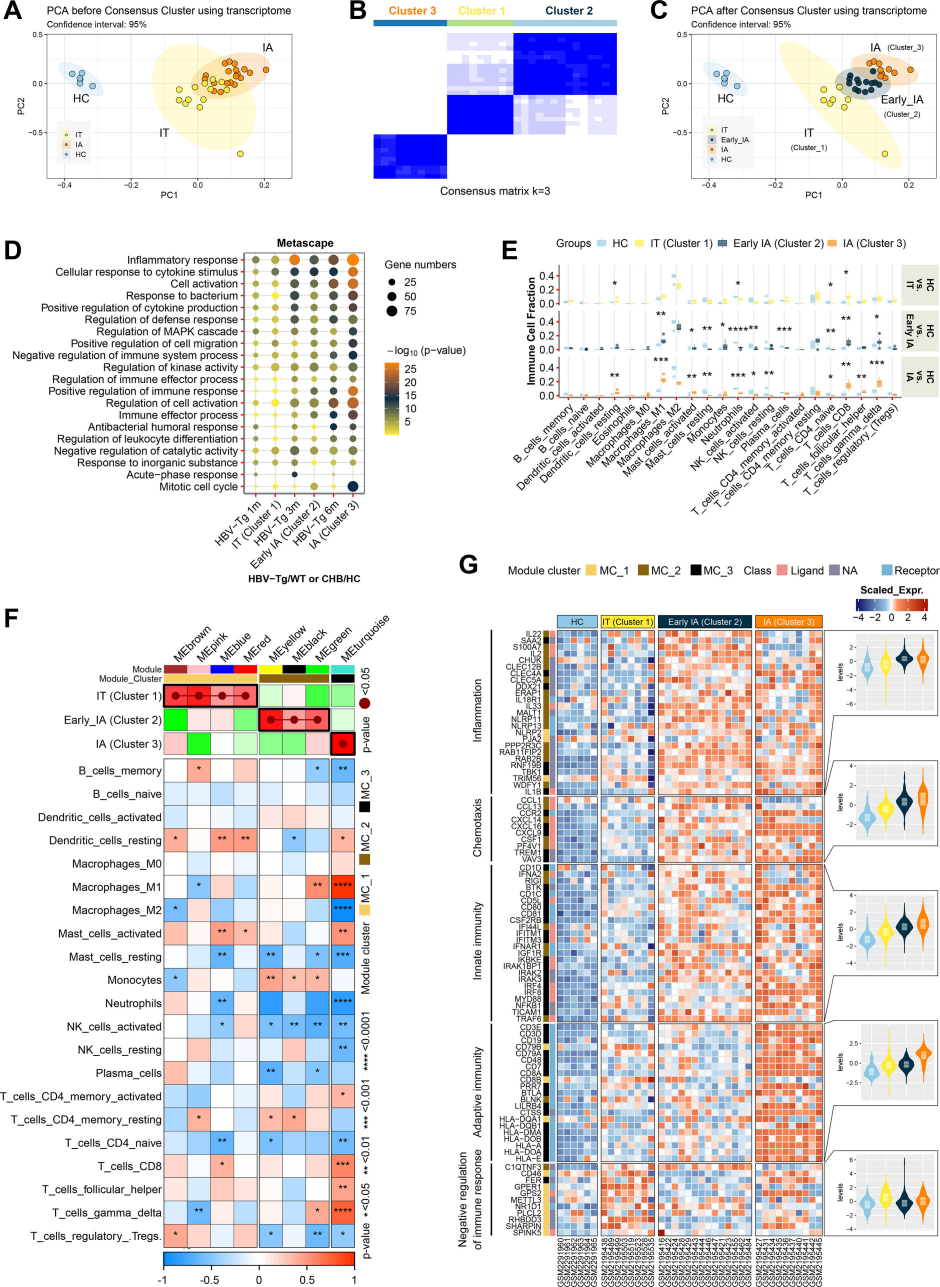
Comparison of hepatic immune-related genes signature between chronic HBV-infected patients and HBV-Tg mice. Hepatic gene expression data of 32 CHB patients and 6 uninfected healthy controls were obtained from GSE83148 and analyzed. (**A**) PCA for HCs group and two CHB patient groups (IT and IA) based on levels of ALT, AST, and HBV DNA. (**B**) Consensus matrix of three clusters (groups) identified optimally among these CHB patients by consensus clustering. (**C**) PCA for HCs group and three CHB patient groups (IT, early-IA, and IA) after consensus clustering. (**D**) Metascape enrichment analysis (GO-BP) for top 500 most upregulated genes in each HBV-Tg or CHB group. (**E**) Intrahepatic infiltration of immune cells was analyzed by CIBERSORT. (**F**) Eight significant IRG modules were identified (from MEbrown to MEturquoise) by WGCNA. IRG modules positively correlated to IT, early-IA, or IA were clustered into MC_1, MC_2, or MC_3. Heat map showed correlations between IRG modules and immune cell fractions analyzed by Pearson correlation analysis. (**G**) Heat map showed expression profiles of representative genes in the module cluster (MC_1, MC_2, or MC_3), including module markers, molecular classifications of encoded proteins, gene function annotation, and the overall expression level of these genes in different groups. Gene expression values were normalized by row (scale). *P < 0.05, **P < 0.01, ***P < 0.001, ****P < 0.0001. early-IA, early stage of immune activation; 1m, 1 month old; 3m, 3 months old; 6m, 6 months old.

Principal component analysis (PCA) depicted clear overlap between samples from IT and IA patients, suggesting a resemblance in hepatic IRG signatures among certain IT and IA patients with varying ALT/AST and HBV DNA levels (Fig. 3A). Consensus clustering was then employed to accurately identify groups of CHB patients based on their hepatic IRG profiles, resulting in the identification of three optimal clusters (groups) among these CHB patients (Fig. 3B). Cluster 1, consisting of seven CHB patients with normal ALT and AST levels and a high HBV-DNA load, was categorized as the IT group. Cluster 3, composed of 11 CHB patients with increased ALT and AST levels and a relatively low HBV-DNA load, was designated as the IA group. Interestingly, cluster 2, positioned between clusters 1 and 3, contained five patients with normal ALT levels and nine with elevated ALT levels, thereby forming a new "early-IA" group (Fig. 3C).

Metascape enrichment analysis was employed to illustrate the enriched pathways of the top 500 upregulated genes from each HBV-Tg mice or CHB patient group concurrently (accounting for homologous genes in humans and mice). The outcomes underscored a significant similarity in the degree of immune/inflammation-related pathway activation between 1-month-old HBV-Tg mice and IT patients, 3-month-old HBV-Tg mice and early-IA patients, and 6-month-old HBV-Tg mice and IA patients (Fig. 3D).

The analysis of hepatic immune cell infiltration was also conducted using CIBERSORT among these CHB patients (Fig. 3E). Analogous to the findings in 3-month-old HBV-Tg mice, early-IA patients exhibited a significant increase in hepatic infiltration of monocytes and macrophages M1 (*P* = 0.034 and *P* = 5.2e−05, respectively) (Fig. 3E). Additionally, using WGCNA, three clusters representing significant IRG modules (in different colors) were positively correlated with the IT, early-IA, or IA groups. Notably, three IRG modules positively correlated with the early-IA group (*P* < 0.05) were also found to be positively correlated with monocytes infiltration (*P* < 0.01, *P* < 0.05, and *P* < 0.05, respectively) (Fig. 3F).

Further insight into the functions of these IRG modules and their expression at different time points was gained through a gene expression heatmap (Fig. 3G), highlighting representative IRGs within gene modules linked to specific immune/inflammation-related biological processes. Similar to 1-month-old HBV-Tg mice, IT patients exhibited increased expression of genes associated with the negative regulation of inflammation and immunity. In parallel, IA patients, akin to 6-month-old HBV-Tg mice, displayed significantly upregulated genes associated with both innate and adaptive immunity. Notably, within the three patient groups, early-IA patients showcased the highest gene expression related to inflammation activation, mirroring the profile observed in 3-month-old HBV-Tg mice (Fig. 3G).

### Enhanced activation of transcription factor Stat3 strongly associated with robust IRGs expression in 3-month-old HBV-Tg mice

The preceding findings have demonstrated that hepatic gene expression in 3-month-old HBV-Tg mice exhibits characteristics resembling the early stage of immuno-inflammation activation, akin to the early-IA CHB patients. Subsequently, our focus shifted to investigating the pivotal TFs responsible for the substantial increase in IRGs expression.

**Fig 4:**
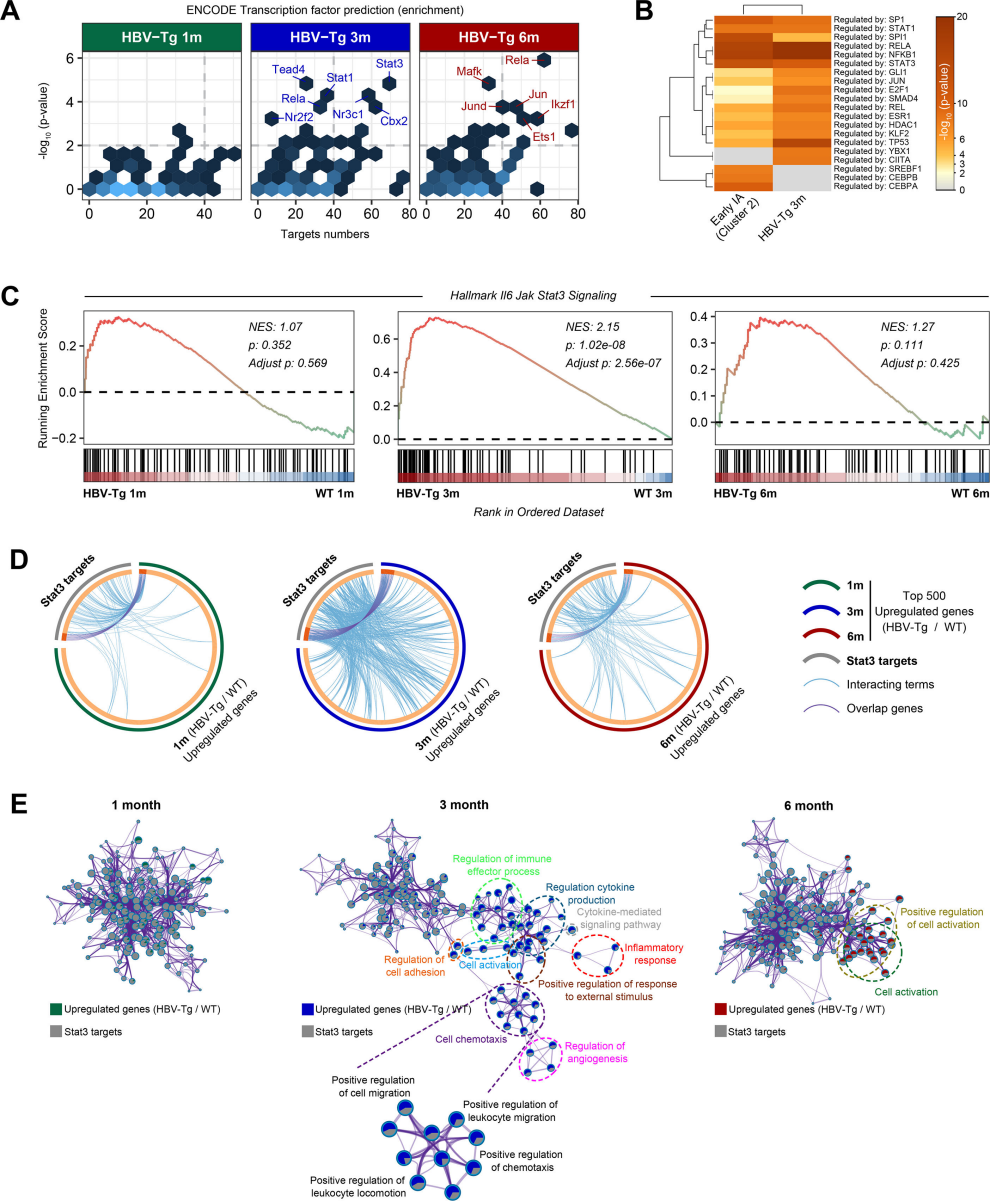
Stat3 was predicted to be the most significantly activated transcription factor in 3-month-old HBV-Tg mice. The top 500 upregulated genes in each HBV-Tg mice group or early-IA CHB patients were used for analysis in this section. (**A**) Transcription factors enrichment analysis by ENCODE database in each HBV-Tg mice group. (**B**) Heat map of transcription factor predicted by TRRUST database in early-IA CHB patients and 3-month-old HBV-Tg mice. (**C**) GSEA analysis with hallmark gene sets of “Il6 Jak Stat3 signaling” in each HBV-Tg mice group. (**D**) Circos plots showed overlaps at gene level (light purple) or term level (light blue) between Stat3 targets and upregulated genes in each HBV-Tg mice group. (**E**) Biological networks showed functional overlaps between Stat3 targets and upregulated genes in each HBV-Tg mice group. 1m, 1 month old; 3m, 3 months old; 6m, 6 months old; early-IA, early stage of immune activation.

Initially, a transcription factor enrichment analysis was conducted using both the ENCODE and TRRUST databases. Transcription factors with a *P*-value below 0.01 and targeting over 40 genes were identified as candidate TFs. The outcomes indicated that Stat3 and Rela (P65) were ranked as the top activated transcription factors in 3-month or 6-month HBV-Tg mice, respectively (Fig. 4A). Furthermore, heatmap visualization demonstrated that Rela and Stat3 were also the predominant enriched TFs both in 3-month HBV-Tg mice and early-IA CHB patients (Fig. 4B). GSEA of hallmark gene sets highlighted a significant activation of “hallmark Il6 Jak Stat3 signaling” in 3-month HBV-Tg mice (*P* = 1.02e−08) (Fig. 4C).

Subsequently, we delved into comprehending the specific role of Stat3 in the immune activation process. A total of 142 high-confidence targets of Stat3 were sourced from the TRRUST database. Overlapping genes and Gene Ontology terms were identified between these Stat3 targets and the top 500 upregulated genes in each HBV-Tg mice group. The Circos plot revealed that compared to 1-month and 6-month HBV-Tg mice, the 3-month HBV-Tg mice exhibited a slightly increased overlap at the gene level (light purple) while displaying a considerably amplified overlap at the term level (light blue) (Fig. 4D). To delve deeper, biological networks were employed to scrutinize and visualize the shared genes and terms (Fig. 4E). Remarkably, the overlapping terms between Stat3 target genes and the top 500 upregulated genes in 3-month HBV-Tg mice encompassed terms such as “inflammatory response,” “cell chemotaxis,” “regulation of cytokine production,” “cell activation,” “regulation of cell adhesion,” among others (Fig. 4E).

**Fig 5:**
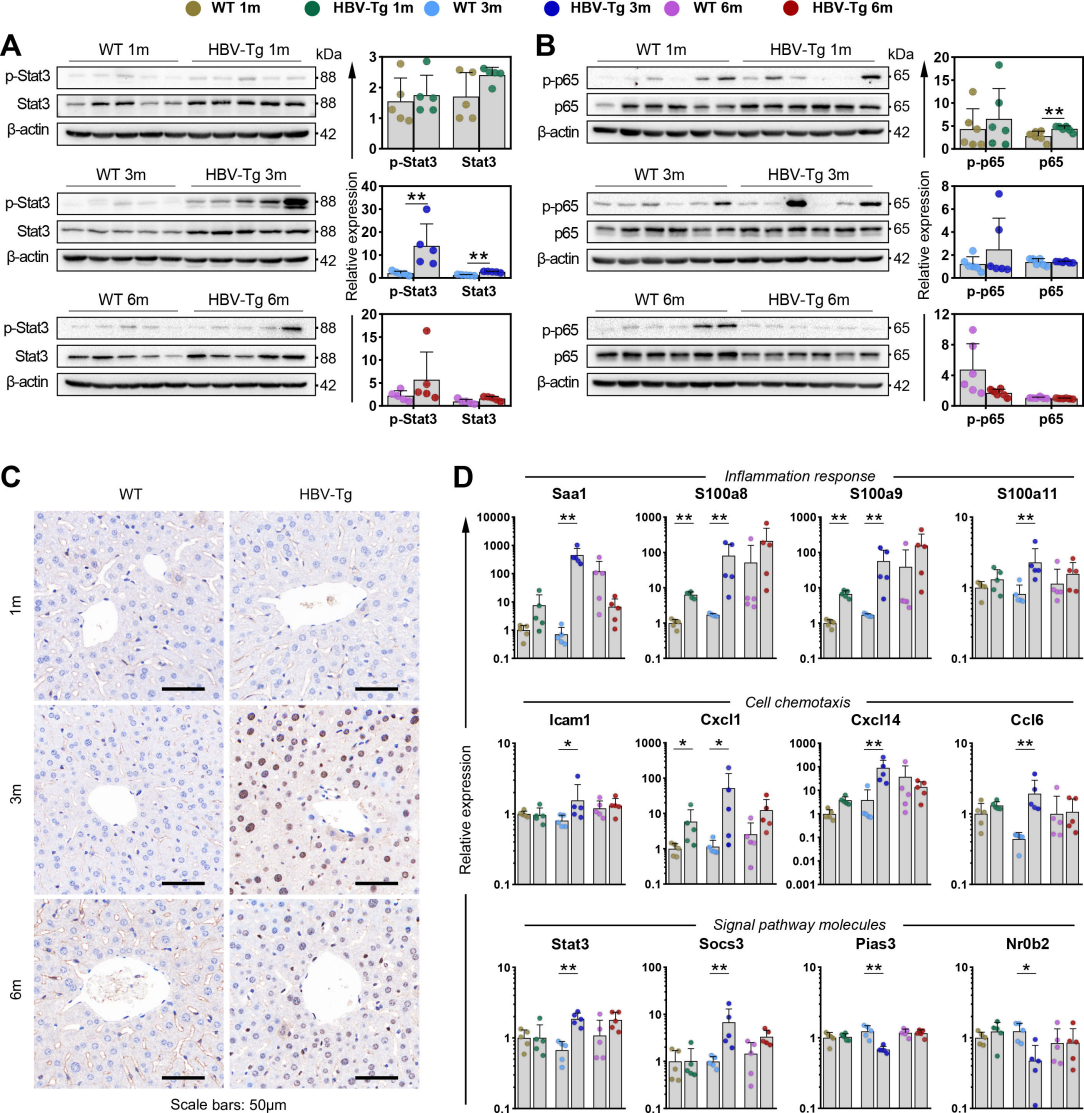
Enhanced expression and activation of transcription factor Stat3 were verified in 3-month-old HBV-Tg mice. Liver tissues were obtained from HBV-Tg and WT mice at 1, 3, and 6 months old for detection in this section (*n* = 5/group). (**A**) Expression of total Stat3 and p-Stat3 was detected by western blot. (**B**) Expression of total P65 and p-P65 was detected by western blot. (**C**) Expression of p-Stat3 was detected in liver sections by immunohistochemical staining (representative images). p-Stat3-positive hepatocyte nuclei were shown in brown. Scale bar: 50 μm. (**D**) Expression of genes related to Stat3 signaling activation was determined by qPCR. These genes were representative genes involving in inflammation response, cell chemotaxis, and regulators of Stat3 signaling pathway. (**A, B, D**) Each dot represented an individual mouse, and each bar summarized a data set using the mean and standard error of the data set. *P < 0.05, **P < 0.01. 1m, 1 month old; 3m, 3 months old; 6m, 6 months old; p-Stat3, phosphorylated Stat3 (pY705).

Subsequent validation was conducted on Stat3 expression and activation in HBV-Tg mice. A notable elevation in protein levels of both total Stat3 and phosphorylated Stat3 (pY705-Stat3) was noted in 3-month HBV-Tg mice (Fig. 5A). Although P65 (Rela) was predicted to be activated in 3-month or 6-month HBV-Tg mice, no significant changes were observed in either total or phosphorylated P65 in these two groups (Fig. 5B). This observation might stem from the overlapping target genes among transcription factors. Additionally, an increased expression of pY705-Stat3 in liver specimens was confirmed in 3-month HBV-Tg mice, predominantly localized within the hepatocyte nucleus (Fig. 5C). Meanwhile, the expression of certain genes linked to Stat3 activation was assessed using quantitative PCR. The results revealed that the expression of inflammatory genes (Saa1, S100a8, S100a9, S100a11) (26, 27) and genes associated with cell migration and chemotaxis (Icam1, Cxcl1, Cxcl14, Ccl6) (28–31) was significantly upregulated at the third month (Fig. 5D). Intriguingly, the inducible negative regulator of Stat3 signaling (Socs3) (32) displayed significant upregulation, while the endogenous suppressors of Stat3 signaling (Pias3 and Nr0b2) (33, 34) were markedly downregulated in 3-month HBV-Tg mice.

### Hepatic p-Stat3 expression positively correlates with serum ALT levels in young CHB patients

**Fig 6:**
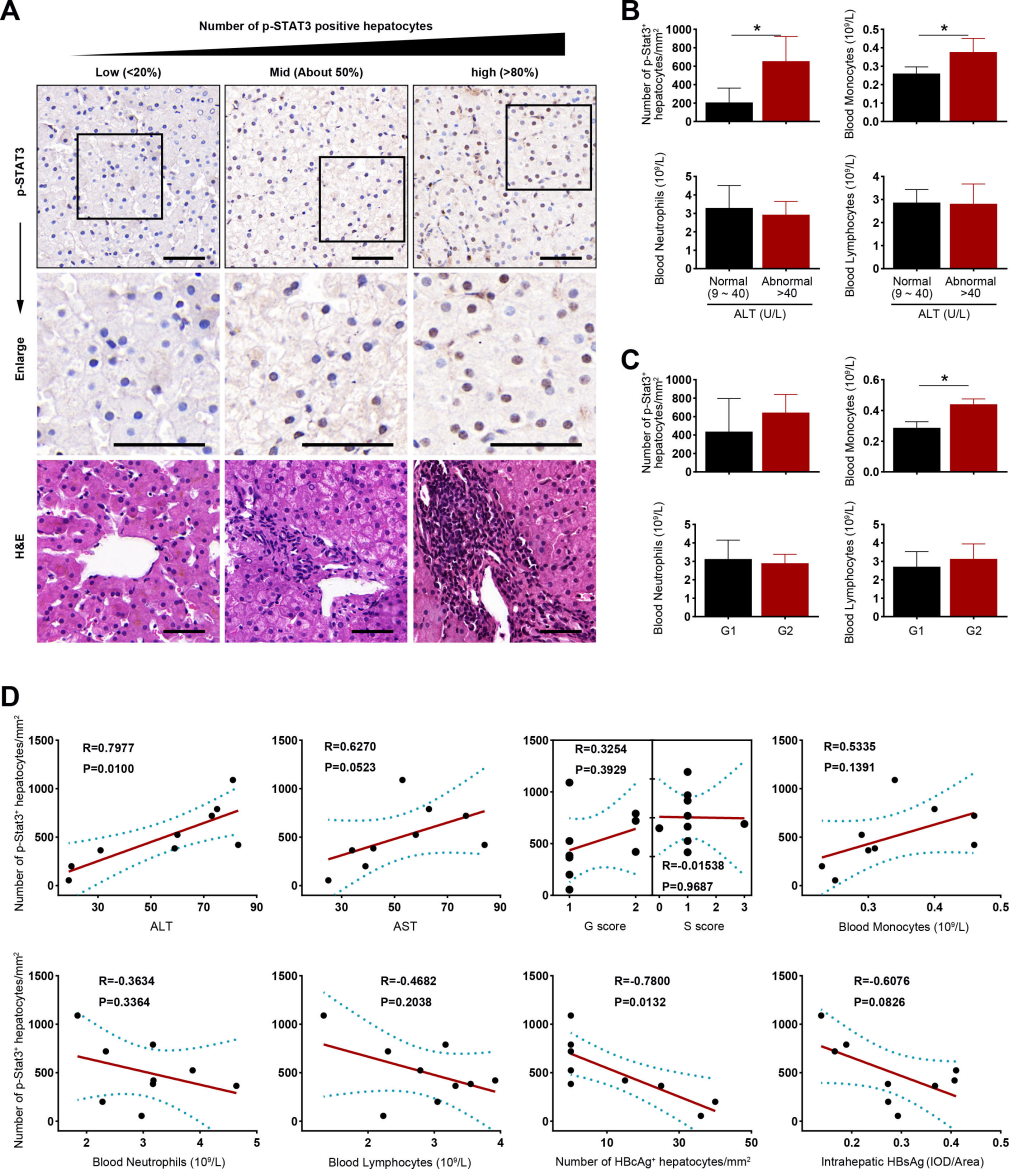
Hepatic p-Stat3 expression in young CHB patients. (**A**) p-Stat3 expression was assayed in the paraffin-embedded liver tissue sections from nine young CHB patients with normal or slightly elevated ALT levels (≤2× ULN) by immunohistochemistry method. Representative images of p-Stat3 staining and corresponding H&E staining from three patients are displayed by the increasing number of p-Stat3-positive hepatocytes from left to right. Positive staining for p-Stat3 was shown brown. Scale bar: 50 µm. (**B, C**) The number of p-Stat3-positive hepatocytes, monocytes, neutrophils, and lymphocytes was compared between patients with normal and abnormal ALTs (**B**) or patients with G1 and G2 stage (**C**). (**D**) Correlation analysis was performed between p-Stat3-positive hepatocytes and serum ALT or AST levels, G/S scores, the number of monocytes, neutrophils, or lymphocytes in the peripheral blood, the number of HBcAg-positive hepatocytes, and the mean optical density (IOD/area) of HBsAg. AST, aspartate aminotransferase; G, inflammatory activity; S, degree of fibrosis; IOD, integrated optical density.

In this context, we proceeded to further assess Stat3 activation during the early inflammatory phase of chronic HBV infection, focusing on paraffin-embedded liver tissues from nine young CHB patients with normal or slightly elevated ALT levels (≤2× ULN). Comprehensive data, including clinical information, laboratory test results, and histopathological examinations, were retrospectively collected for these cases (Table 1). Staining for p-Stat3 positivity predominantly localized within the nuclei of hepatocytes, and notably, a higher abundance of p-Stat3-positive hepatocytes was evident in liver tissue samples from patients with more pronounced liver inflammation (Fig. 6; Fig. S1). Patients exhibiting elevated ALT levels (>40 U/L) displayed a greater number of p-Stat3-positive hepatocytes and blood monocytes compared to those with normal ALT levels (9–40 U/L) (both *P* < 0.05) (Fig. 6B and C). Subsequent correlation analysis revealed a robust positive correlation between serum ALT levels and the count of p-Stat3-positive hepatocytes (*R* = 0.7977, *P* = 0.0100), in contrast to AST levels (*R* = 0.6270, *P* = 0.0523), G score (*R* = 0.3254, *P* = 0.3929), or S score (*R* = −0.01538, *P* = 0.9687) (Fig. 6D). Moreover, the count of p-Stat3-positive hepatocytes exhibited an upward trend with increasing numbers of monocytes, accompanied by decreasing trends in neutrophils and lymphocytes. Notably, the expression levels of either HBcAg or HBsAg exhibited a negative correlation with the count of p-Stat3-positive hepatocytes (HBcAg: *R* = −0.7800, *P* = 0.0132; HBsAg: *R* = −0.6076, *P* = 0.0826) (Fig. 6D).

**TABLE 1:** Clinical and laboratory characteristics of CHB patients (for hepatic p-Stat3 detection on the paraffin-embedded liver tissue section)

Patient ID	P10301	P02293	P09227	P09516	P05357	P11845	P04089	P08063	P01592
Age (years)	12	3	3	3	10	3	4	16	3
Sex	Female	Female	Male	Female	Female	Male	Male	Male	Male
WBC (10^9^/L)	5.57	5.68	8.42	7.27	7.89	5.22	7.23	3.89	7.77
Platelets (10^9^/L)	272	306	224	310	239	115	252	174	296
Lymphocytes (10^9^/L)	2.23	3.05	3.32	3.55	2.79	2.3	3.17	1.33	3.92
Neutrophils (10^9^/L)	2.97	2.28	4.64	3.17	3.87	2.34	3.17	1.84	3.18
Monocytes (10^9^/L)	0.25	0.23	0.3	0.31	0.29	0.46	0.4	0.34	0.46
ALT (U/L)	19	20	31	59	60	73	75	81	83
AST (U/L)	25	39	34	42	58	77	63	53	84
HBsAg	+	+	+	+	+	+	+	+	+
HBeAg	+	+	+	+	+	+	+	+	+
HBV DNA (log_10_ IU/mL)	8.26	8.36	8.11	8.49	6.08	8.46	7.19	7.99	6.96
Inflammation grading (G)/fibrosis staging (S) score	G1/S1	G1/S1	G1/S0	G1/S1	G1/S1	G2/S1	G2/S1	G1/S1	G2/S3

### Inhibition of Stat3 activation significantly attenuates intrahepatic immune/inflammation development in HBV-Tg mice

**Fig 7:**
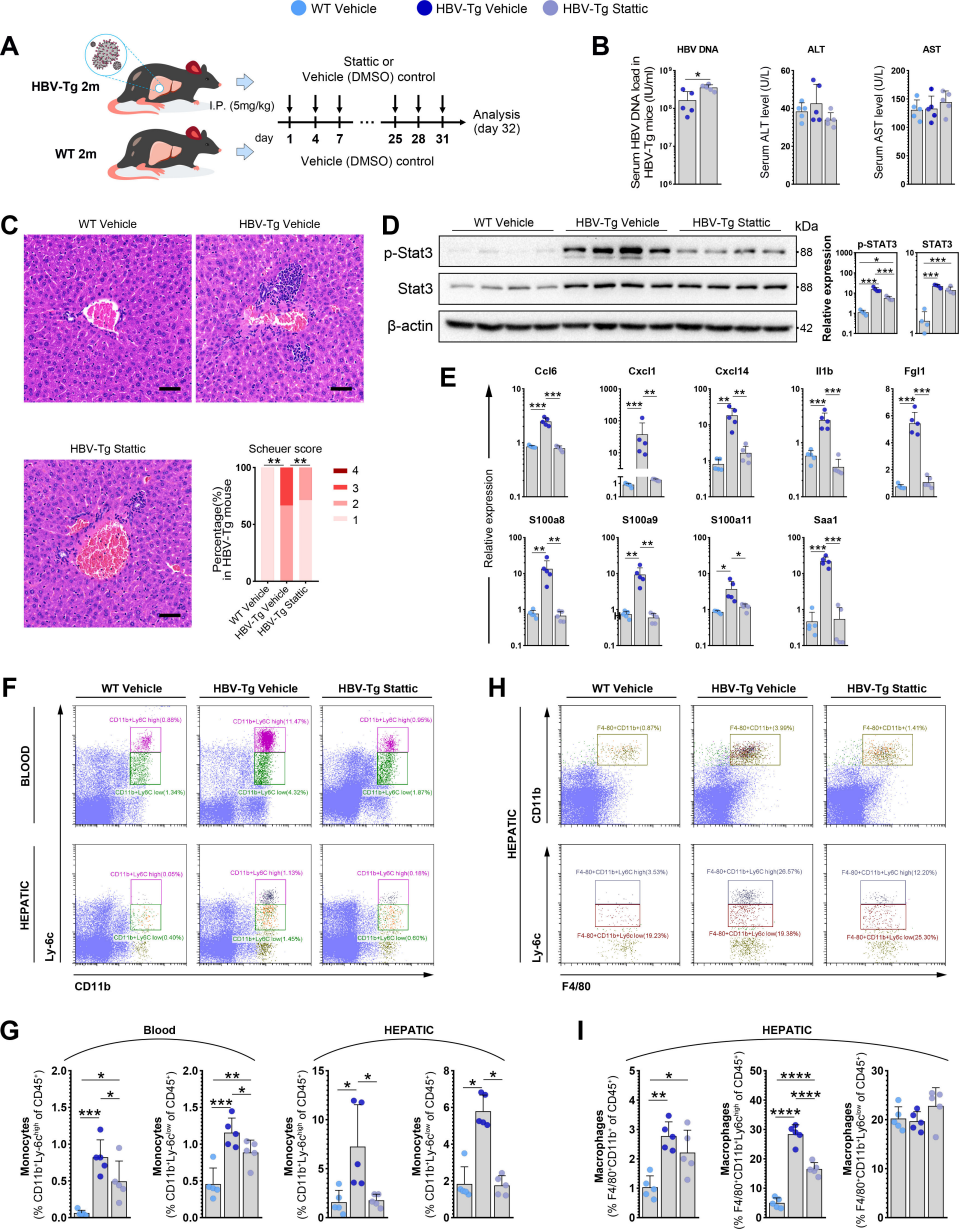
*In vivo* efficacy of inhibiting Stat3 activation with selective Stat3 inhibitor, Stattic, in HBV-Tg mice. (**A**) Two-month-old HBV-Tg or WT mice were treated with Stat3 inhibitor Stattic or Vehicle for 1 month as indicated in experimental procedure plot (*n* = 5/group). (**B**) Serum levels of HBV DNA, ALT, and AST in groups of HBV-Tg Stattic, HBV-Tg Vehicle, or WT Vehicle. (**C**) Representative images of H&E staining of liver tissue sections. The inflammation grading in liver sections was evaluated with Scheuer scoring systems (Scheuer score). Scale bar: 50 µm. (**D**) Expression of total Stat3 and p-Stat3 in liver tissue was detected by western blot. (**E**) Expression of inflammation-related genes in liver tissue was determined by qPCR. (**F**) Representative flow cytometry plots of CD11b^+^Ly-6c^high^ and CD11b^+^Ly-6c^low^ monocyte in peripheral blood and liver tissue. (**G**) Frequencies of CD11b^+^Ly-6c^high^ and CD11b^+^Ly-6c^low^ monocyte in peripheral blood and liver tissue were expressed as the percentage in CD45^+^ leukocytes. (**H**) Representative flow cytometry plots of hepatic macrophage (F4/80^+^CD11b^+^) and monocytes-derived macrophages (F4/80^+^CD11b^+^Ly-6c^high^ and F4/80^+^CD11b^+^Ly-6c^low^). (**I**) Proportions of hepatic F4/80^+^CD11b^+^ macrophages, F4/80^+^CD11b^+^Ly-6c^high^ and F4/80^+^CD11b^+^Ly-6c^low^ monocytes-derived macrophages in CD45^+^ leukocytes. (B, D, E, G, I,) Values for individual mice were given, as well as mean ± standard error. *P < 0.05, **P < 0.01, ***P < 0.001, ****P < 0.0001. Stattic, a small molecule inhibitor of Stat3; Vehicle, solvent control; Schuer score, an inflammation scale.

Based on the findings elucidated above, we hypothesized that the activation of Stat3 might play a pivotal role in triggering hepatic inflammation during the chronic HBV carrier state. To validate this hypothesis, we investigated the *in vivo* efficacy of dampening Stat3 activation through the selective Stat3 inhibitor, Stattic, in HBV-Tg mice (Fig. 7A).

In comparison to the HBV-Tg vehicle control group, the HBV-Tg Stattic group exhibited a notably higher level of serum HBV DNA (*P* < 0.05) while demonstrating similar levels of ALT and AST (Fig. 7B). Histological analysis of liver specimens from the HBV-Tg Stattic group revealed a significantly diminished degree of inflammatory infiltration (Scheuer score 1–2) as opposed to the HBV-Tg vehicle controls (Scheuer score 2–3) (Fig. 7C).

Notably, Stattic treatment led to a substantial reduction in the levels of Stat3 activation (p-Stat3) without causing significant changes in total Stat3 levels in the HBV-Tg Stattic group when compared to the HBV-Tg vehicle control group (Fig. 7D). Importantly, the expression of several inflammation-related genes, which were upregulated in HBV-Tg vehicle mice, experienced a considerable decline following Stattic treatment. These genes included Il1b, S100a8, Ccl6, Cxcl1, Cxcl14, among others (all *P* < 0.05) (Fig. 7E).

Given our previous observation of enhanced intrahepatic monocyte infiltration during the initial stages of immune/inflammation activation in both HBV-Tg mice and CHB patients, we subsequently explored the influence of Stat3 inhibition on intrahepatic monocyte infiltration. Our results indicated that the proportions of both inflammatory monocytes (CD11b^+^Ly-6c^high^) and regulatory monocytes (CD11b^+^Ly-6c^low^) were markedly higher in peripheral blood and liver tissues of the HBV-Tg vehicle group compared to the WT vehicle group (all *P* < 0.05) (Fig. 7F and G). Importantly, treatment with Stattic led to a significant reduction in the proportions of both peripheral and hepatic monocytes in the HBV-Tg Stattic group (all *P* < 0.05) (Fig. 7F and G).

Furthermore, considering that liver-infiltrating monocytes are capable of differentiating into hepatic macrophages (F4/80^+^CD11b^+^Ly-6c^+^) (35), we analyzed the frequencies of these macrophage subtypes between the HBV-Tg Stattic and control groups. Significantly higher proportions of macrophages (F4/80^+^CD11b^+^) and inflammatory monocyte-derived macrophages (F4/80^+^CD11b^+^Ly-6c^high^) were observed in the HBV-Tg vehicle group compared to the WT vehicle group (*P* < 0.01 and *P* < 0.0001) (Fig. 7H and I). Notably, Stattic treatment effectively reduced the proportion of inflammatory monocyte-derived macrophages (F4/80^+^CD11b^+^Ly-6c^high^) in the HBV-Tg Stattic group (*P* < 0.0001) (Fig. 7H and I). However, no significant differences were observed in F4/80^+^CD11b^+^Ly-6c^low^ macrophages among these groups (Fig. 7H and I).

## DISCUSSION

Despite some existing research reporting the existence of inflammatory foci and hepatocyte damage in liver biopsies from certain chronic hepatitis B patients during the “immune tolerance” phase (36–38), the precise immune characteristics and specific initiating factors of this early immune activation stage have remained largely unexplored. In this investigation, utilizing an HBV-Tg mouse model characterized by elevated HBV-DNA levels and HBeAg expression, we observed a gradual escalation of liver injury and immune cell infiltration over the course of 3 and 6 months, in contrast to HBV-Tg 1-month and WT mice. Remarkably, enhanced liver inflammation has already occurred despite the presence of normal ALT levels in 3-month-old HBV-Tg mice. These pathological and serological observations collectively indicate a transition from immune tolerance to immune activation in these HBV-Tg mice, with the early phases of immune activation potentially occurring at 3 months of age. Furthermore, our transcriptomic analysis of HBV-Tg mice unveiled an absence of significant changes in immune/inflammation-related genes at 1 month, followed by a noteworthy upregulation of innate immune and inflammation-associated genes, alongside increased monocyte infiltration at 3 months. These findings not only underscore the presence of early-stage HBV-related immune activation in 3-month-old HBV-Tg mice but also shed light on the pivotal role of innate immunity in triggering this immune response.

Nowadays, modern algorithms have been widely utilized to discover novel genes or mechanisms involved in disease pathogenesis. In our study, the integration of bioinformatics analysis hinted transcription factor Stat3 might be the potential key factor triggering HBV-induced liver inflammation and helped us to conduct subsequent validation experiments. Then, increased Stat3 activation was verified in early stage of HBV-induced liver inflammation using liver tissues from 3-month-old HBV-Tg mice and chronic HBV-infected children with relatively higher ALT levels. Finally, we performed *in vivo* Stat3 inhibition experiment and further confirmed high levels of intrahepatic Stat3 activation as a major contributor to the shift from HBV-related immune tolerance to immune activation. Considering that HBV-Tg mice are immunologically tolerant to HBV antigens, these results also suggest that factors beyond HBV-specific immunity can initiate immune-mediated inflammation in chronic HBV carriers (39, 40).

Liver inflammation serves as a hallmark of the activation of either non-specific or specific immune responses to HBV infection and is a critical parameter for assessing the need for antiviral therapy. In clinical practice, alanine aminotransferase is frequently utilized as a surrogate marker to evaluate hepatic inflammation in lieu of liver biopsy. Nevertheless, the reliance on ALT levels to gauge the extent of liver inflammation is becoming increasingly intricate (41–44), particularly in the context of chronic hepatitis B patients in the immune tolerance phase (36–38). Conventionally, patients in the IT phase were assumed to exhibit quiescent histological activity, a low risk of disease progression, and limited responsiveness to antiviral interventions. Consequently, guidelines typically did not advocate antiviral treatment for this category of patients. However, recent investigations have challenged this paradigm by revealing that substantial liver inflammation can indeed manifest during the IT phase despite normal ALT levels (36–38). These reports imply the potential existence of an intermediate stage bridging the conventional "IT" and "immune activation" phases. In our study, through a longitudinal analysis of the hepatic transcriptome in HBV-Tg mice and CHB patients, we successfully identified this transitional stage characterized by heightened expression of inflammation-related genes and enhanced monocyte infiltration, all while maintaining normal ALT levels.

It was previously postulated that inflammatory mediators originating from HBV-specific immune responses targeting HBV-infected hepatocytes, further bolstered by non-specific immunity, underlay the pathogenesis of chronic hepatitis B, particularly during the immune activation phase (45–49). Nonetheless, investigations focusing on neonates, children, or young adults with chronic HBV infection have unveiled a more intricate picture. These studies demonstrated the presence of HBV-specific T cells producing Th1-type cytokines, even during the immune tolerance phase, often exhibiting better quantity and functionality compared to the immune activation phase (4). Remarkably, HBV-specific T cell clones, despite being tolerated, endure in fully developed transgenic mice exposed to HBV antigen at birth (50, 51). These collective findings challenge the notion that HBV-specific CD8^+^ T cells solely act as initiators of the transition from immune tolerance to immune activation. Thus, it is plausible that a broader array of mechanisms contributes to the orchestration of specific and non-specific immune and inflammatory responses during this transitional phase.

Unveiling the factors responsible for the transition from immune tolerance to immune activation presents a considerable challenge due to the practical difficulties in procuring liver biopsies from patients in the actual immune tolerance and early immune activation phases. Although there is a lack of small animal models that reproduce human-like HBV infections, mouse models of HBV replication have been widely used for HBV research. Therefore, we employed an HBV-Tg mouse model with high levels of viral replication and spontaneous liver inflammation, facilitating dynamic sampling and direct observation of liver tissue changes. This approach enabled the identification of distinct stages within the HBV-Tg mice at different ages, namely the “stage with no signs of hepatic inflammation” (1 month), the “early stage of inflammatory responses” (3 months), and the “stage of prolonged inflammation” (6 months). The comprehensive gene expression profiles for each stage revealed a marked surge in acute inflammation-related genes and the infiltration of monocytes solely during the early stage of inflammatory responses. Intriguingly, a parallel early immune activation stage with gene profiles akin to those observed in the HBV-Tg mice during the “early stage of inflammation” was also recognized in patients with chronic HBV infection through analysis of the GSE data sets. Collectively, our findings suggest that the conversion from a non-inflammatory phase to an inflammatory phase may be instigated by inflammation or innate immune activation.

Importantly, our study successfully pinpointed the pivotal upstream transcription factor, Stat3, linked to these alterations in gene expression during the early inflammation activation stage. Notably, the inhibition of Stat3 activation yielded significant mitigation in the degree of infiltration by inflammatory cells and hepatocyte injury. This inhibition also resulted in reduced expression levels of acute inflammation-related genes (such as S100a8, S100a9, Cxcl1, or Cxcl14) and a decline in the proportions of infiltrating inflammatory monocytes and macrophages.

Prior research has underscored the pivotal role of Stat3 signaling activation in diverse liver physiological and pathological processes, encompassing liver regeneration (52), chronic liver inflammation (53), liver fibrosis (54), and hepatocellular carcinoma (55). Alongside the conventional cytokines and growth factors that have been previously identified as Stat3 activators, recent studies have unveiled the involvement of neuroendocrine factors and ischemic/hypoxic stimuli in Stat3 activation. Our own findings draw attention to a noteworthy observation: a significant reduction in mitochondrial function coincided with the onset of hepatic inflammation during the early stages of inflammatory responses. It is worthwhile to investigate the relationship between mitochondrial stress and Stat3 activation in the future.

**Fig 8:**
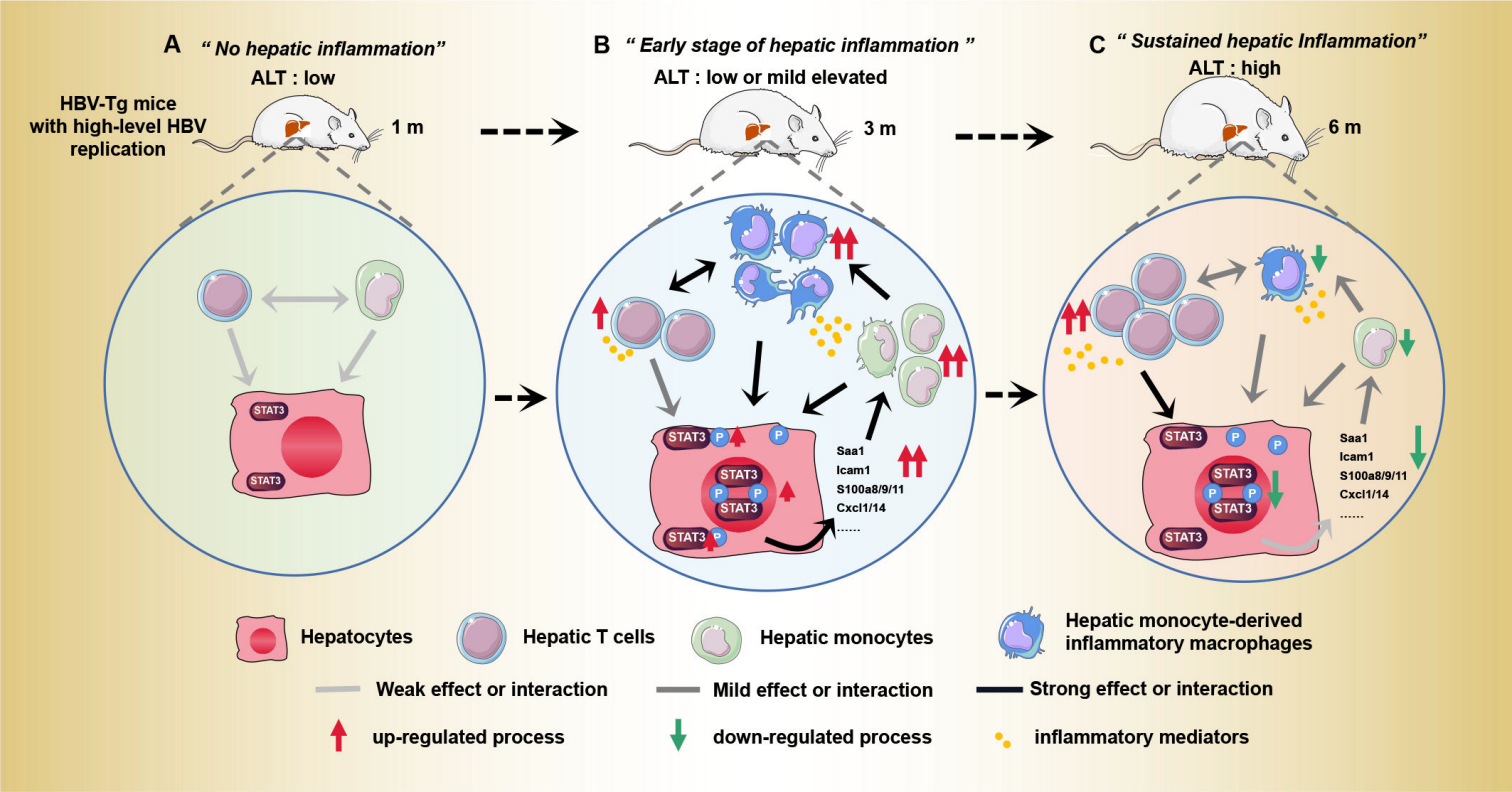
Proposed model for the initiation role of Stat3 activation in HBV-induced hepatic inflammation. (A) HBV-Tg mice with high-level HBV replication had no obvious hepatic inflammation and low ALT levels at 1 month (m). (B) Stat3 was activated during the early stage of hepatic inflammation with increasing age. Activation of Stat3 induced a surge of acute inflammatory factors (such as Saa1, Icam1, S100a8/9/11, Cxcl1/14, et al.), followed by dramatically increased monocytes/macrophages and mildly increased T cells in the liver. In this stage, minor liver inflammation or mildly elevated ALT levels might be observed. (C) After that, Stat3 activity declined, followed by gradually decreased acute inflammation-associated factors and monocyte/macrophages in liver. Meanwhile, T cell activity was further enhanced, which promoted the progression of liver inflammation and injury.

In conclusion, our study illuminates Stat3 as a key factor contributing to HBV-induced hepatic inflammation activation (Fig. 8). These findings furnish novel insights into comprehending the mechanisms of transition from immune-tolerant phase to immune-active phase in patients with chronic HBV infection. The main limitations of our study are that HBV-Tg mice are not naturally infected with HBV and lack the early steps of HBV infection (viral entry and cccDNA formation). Therefore, the conclusions should be further confirmed in more HBV infection or replication animal models, or a large chronic HBV-infected patient cohort.

## AFTER EPUB

*[This article was published on 5 March 2024 with missing affiliations for the first two authors. The affiliations were corrected in the current version, posted on 21 March 2024.]*

## SUPPLEMENTAL MATERIAL

Fig. S1 mbio.03068-23-s0001.pdf Representative images of H&E staining and immunohistochemistry (including p-Stat3 staining, HBcAg staining, and HBsAg staining) of all 9 young CHB patients.

Tables S1 and S2 mbio.03068-23-s0002.doc IRGs obtained from the AmiGO 2 and primers used in the quantitative real-time PCR analysis.

Supplemental text mbio.03068-23-s0003.doc List of reagents, animal models, human samples, instruments, and software.

Table S3 mbio.03068-23-s0004.xls Differential analysis of mRNA expression levels between HBV-Tg and WT mice.

Table S4 mbio.03068-23-s0005.xls Gene set enrichment analysis of mRNA expression differences between HBV-Tg and WT mice.

ASM does not own the copyrights to Supplemental Material that may be linked to, or accessed through, an article. The authors have granted ASM a non-exclusive, world-wide license to publish the Supplemental Material files. Please contact the corresponding author directly for reuse.
