# Supplementary material for: Stat3 activation-triggered transcriptional networks govern the early stage of HBV-induced hepatic inflammation
Source: mBio. 2024 Mar 5;15(4):e03068-23. doi: 10.1128/mbio.03068-23 (PMC11005361; doi:10.1128/mbio.03068-23)
Supplement: Fig. S1 — Representative images of H&E staining and immunohistochemistry (including p-Stat3 staining, HBcAg staining, and HBsAg staining) of all 9 young CHB patients. [file mbio.03068-23-s0001.pdf]

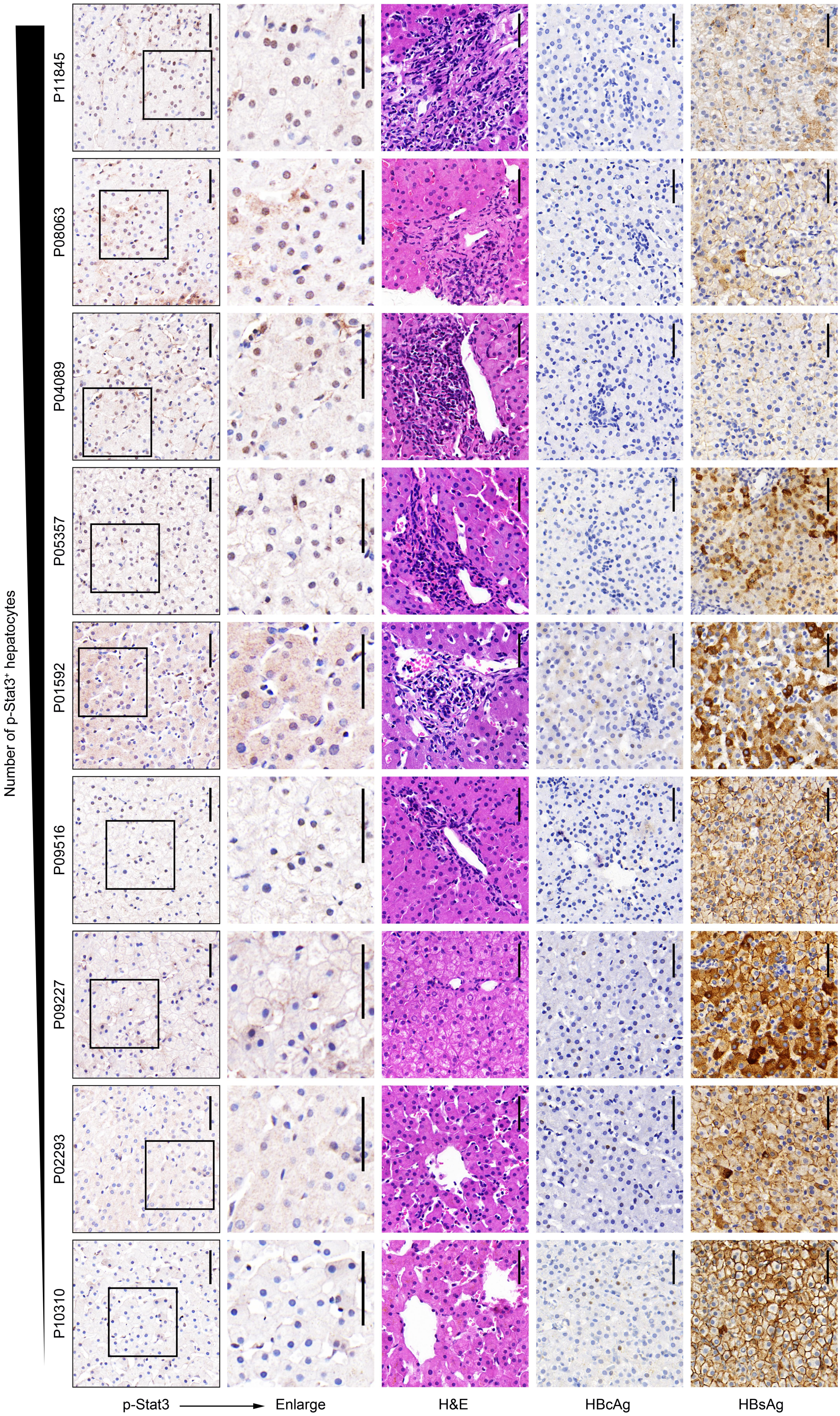
Number of p-Stat3⁺ hepatocytes
P11845
P08063
P04089
P05357
P01592
P09516
P09227
P02293
P10310
p-Stat3
Enlarge
H&E
HBcAg
HBsAg

Fig. S1. Representative images of H&E staining and immunohistochemistry (including p-Stat3 staining, HBcAg staining, and HBsAg staining) of all 9 young CHB patients.

Images are displayed by the increasing number of p-Stat3-positive hepatocytes from left to right. Positive staining of immunohistochemical staining are shown brown-yellow. Typical portal area inflammation of H&E staining are marked by black rectangles and magnified.
