# Supplementary material for: Stat3 activation-triggered transcriptional networks govern the early stage of HBV-induced hepatic inflammation
Source: mBio. 2024 Mar 5;15(4):e03068-23. doi: 10.1128/mbio.03068-23 (PMC11005361; doi:10.1128/mbio.03068-23)
Supplement: Tables S1 and S2 — IRGs obtained from the AmiGO 2 and primers used in the quantitative real-time PCR analysis. [file mbio.03068-23-s0002.doc]

**Table S1. IRGs obtained from the AmiGO 2 (http://amigo.geneontology.org/amigo/landing) database**

| **IRGs for mice (2089)** | **IRGs for human (2274)** |
| --- | --- |
| A2m，Abcc1，Abhd12，Abl1，Abr，Ace，Acer3，Ackr1，Ackr2，Ackr3，Ackr4，Acod1，Acp5，Actg1，Actr2，Actr3，Acvr1，Ada，Adam15，Adam17，Adam8，Adamts12，Adar，Adcy1，Adcy7，Adcy8，Adcyap1，Adgrb1，Adgre1，Adipoq，Adm，Adora1，Adora2a，Adora2b，Adora3，Adra2a，Adrb2，Afap1l2，Ager，Agt，Agtr1a，Agtr1b，Agtr2，Ahcy，Ahcyl，Ahr，Ahsg，Aicda，Aif1，Aim2，Aimp1，Aire，Ak7，Akap1，Akap8，Akirin2，Akna，Akt1，Alcam，Aldh2，Alox15，Alox5，Alox5ap，Alpk1，Ang，Ang2，Ang3，Ang4，Ang6，Ankrd17，Ano6，Anxa1，Anxa3，Aoah，Aoc3，Ap1g1，Ap3b1，Apbb1ip，Apcs，Aplf，Apoa1，Apoa4，Apobec3，Apoe，App，Appl1，Appl2，Aqp4，Arel1，Arf6，Arg1，Arg2，Arhgef2，Arid5a，Arl8b，Arrb2，Ash1l，Atad5，Atg12，Atg5，Atg9a，Atm，Atp7a，Atrn，Axl，AY761185，Azgp1，B2m，B4galt1，Bach2，Bag6，Banf1，Bap1，Batf，Bax，BC051665，Bcap31，Bcar1，Bcl10，Bcl2，Bcl2a1d，Bcl3，Bcl6，Bcl6b，Bcr，Bdkrb1，Bdkrb2，Blk，Blnk，Bmi1，Bmp2，Bmp6，Bmpr1a，Bmpr1b，Bmx，Bpgm，Bpi，Bpifa1，Bpifa5，Bpifb1，Bpifb3，Braf，Brd4，Bst1，Bst2，Btk，Btla，Btn1a1，Btn2a2，Btnl1，Btnl10，Btnl2，Btnl9，C1qa，C1qb，C1qbp，C1qc，C1qtnf12，C1qtnf3，C1ra，C1rb，C1rl，C1s1，C1s2，C2，C2cd4a，C2cd4b，C3，C3ar1，C4b，C4bp，C5ar1，C5ar2，C6，C7，C8a，C8b，C8g，C9，C9orf72，Cacnb3，Cacnb4，Cactin，Cadm1，Calca，Calcoco2，Calcrl，Camk1d，Camk2n1，Camk4，Camp，Capg，Card11，Card9，Carmil2，Casp1，Casp12，Casp4，Casp6，Casp7，Casp8，Cav1，Cblb，Ccdc92，Ccl1，Ccl11，Ccl12，Ccl17，Ccl19，Ccl2，Ccl20，Ccl21a，Ccl21b，Ccl21c，Ccl22，Ccl24，Ccl25，Ccl26，Ccl27a，Ccl27b，Ccl28，Ccl3，Ccl4，Ccl5，Ccl6，Ccl7，Ccl8，Ccl9，Ccn3，Ccn4，Ccr1，Ccr10，Ccr1l1，Ccr2，Ccr3，Ccr4，Ccr5，Ccr6，Ccr7，Ccr8，Ccr9，Ccrl2，Cd14，Cd160，Cd163，Cd177，Cd180，Cd19，Cd1d1，Cd2，Cd200，Cd200r1，Cd200r2，Cd200r3，Cd200r4，Cd22，Cd226，Cd244a，Cd247，Cd24a，Cd274，Cd276，Cd28，Cd300a，Cd300ld3，Cd300lf，Cd33，Cd36，Cd37，Cd38，Cd3d，Cd3e，Cd3g，Cd4，Cd40，Cd40lg，Cd44，Cd46，Cd47，Cd48，Cd55，Cd55b，Cd59a，Cd59b，Cd5l，Cd6，Cd68，Cd7，Cd70，Cd74，Cd79a，Cd79b，Cd80，Cd81，Cd84，Cd86，Cd8a，Cd8b1，Cd96，Cdc37，Cdc42，Cdc42ep2，Cdc42ep4，Cdh17，Cdh5，Cdk19，Ceacam1，Cebpa，Cebpb，Cebpg，Cela1，Celf1，Cers6，Cfb，Cfd，Cfh，Cfhr1，Cfhr2，Cfhr4，Cfi，Cfp，Cgas，Ch25h，Chga，Chia1，Chid1，Chil1，Chil3，Chil4，Chil5，Chil6，Chrna7，Chst1，Chst2，Chst4，Ciita，Cited1，Clcf1，Clec10a，Clec12a，Clec12b，Clec2d，Clec2i，Clec4a1，Clec4a2，Clec4a3，Clec4a4，Clec4b1，Clec4d，Clec4e，Clec4g，Clec4n，Clec5a，Clnk，Clock，Clpb，Clu，Cma1，Cmklr1，Cmtm3，Cnot7，Cnpy3，Cnr1，Cnr2，Cntf，Cntnap2，Col3a1，Colec10，Colec11，Colec12，Coro1a，Cplx2，Cr1l，Cr2，Cracr2a，Crcp，Crh，Crhbp，Crhr1，Crk，Crkl，Crlf2，Crp，Crtam，Csf1，Csf1r，Csf2，Csf2rb，Csf2rb2，Csf3，Csk，Csmd3，Cspg4，Csrp3，Cst7，Cst9，Ctla2a，Ctla4，Ctnnbip1，Cts3，Cts6，Cts7，Cts8，Ctsc，Ctsg，Ctsh，Ctsj，Ctsk，Ctsl，Ctsll3，Ctsm，Ctsq，Ctsr，Ctss，Cuedc2，Cul3，Cx3cl1，Cx3cr1，Cxcl1，Cxcl10，Cxcl12，Cxcl13，Cxcl14，Cxcl15，Cxcl16，Cxcl17，Cxcl2，Cxcl3，Cxcl5，Cxcl9，Cxcr1，Cxcr2，Cxcr3，Cxcr4，Cxcr5，Cxcr6，Cyba，Cybb，Cybc1，Cyld，Cyp19a1，Cyp26b1，Cyp27b1，Cyrib，Cysltr1，D1Pas1，D6Wsu163e，Dab2ip，Dagla，Daglb，Dapk1，Dapk3，Dbnl，Dclre1c，Dcst1，Ddt，Ddx1，Ddx21，Ddx3x，Ddx58，Ddx60，Defa1，Defa10，Defa11，Defa13，Defa14，Defa15，Defa16，Defa17，Defa2，Defa20，Defa21，Defa22，Defa23，Defa24，Defa25，Defa26，Defa27，Defa28，Defa29，Defa3，Defa30，Defa31，Defa34，Defa35，Defa37，Defa39，Defa4，Defa40，Defa41，Defa5，Defa6，Defa7，Defa8，Defa9，Defa-rs10，Defa-rs12，Defa-rs2，Defb1，Defb10，Defb11，Defb12，Defb15，Defb18，Defb19，Defb2，Defb20，Defb21，Defb22，Defb25，Defb29，Defb30，Defb34，Defb35，Defb36，Defb37，Defb38，Defb39，Defb40，Defb41，Defb43，Defb9，Dennd1b，Dgkz，Dhrs7b，Dhx15，Dhx36，Dhx58，Dhx9，Dicer1，Dlg1，Dll1，Dmbt1，Dnaja3，Dnase1，Dnase1l3，Dnase2a，Dock10，Dock11，Dock2，Dpep1，Dpp4，Drd2，Drosha，Dtx3l，Duoxa1，Duoxa2，Dusp10，Dusp22，Dusp3，Dysf，Ear1，Ear10，Ear14，Ear2，Ear3，Ear4，Ear6，Ecm1，Ecsit，Eda，Ednra，Ednrb，Eif2ak1，Eif2ak2，Eif2ak4，Eif2b1，Eif2b2，Eif2b3，Eif2b4，Eif2b5，Elane，Elf1，Elf3，Elf4，Emp2，Endod1，Endou，Enpp1，Enpp2，Enpp3，Entpd7，Eomes，Epg5，Epha2，Ephb2，Ephb6，Ephx2，Eprs，Epx，Erap1，Erbin，Ercc1，Ereg，Ermap，Esr1，Ets1，Evl，Evpl，Exo1，Exosc3，Exosc6，Ext1，Ezr，F12，F2，F2r，F2rl1，F3，F630003A18Rik，F7，F8，Fabp4，Fadd，Fam3a，Fanca，Fancd2，Fas，Fasl，Fasn，Fau，Fbxo38，Fbxo9，Fcamr，Fcer1a，Fcer1g，Fcer2a，Fcgr1，Fcgr2b，Fcgr3，Fcgr4，Fcgrt，Fcho1，Fcna，Fcnb，Fcrl5，Fcrl6，Fcrlb，Fem1a，Fem1al，Fer，Fer1l5，Fes，Ffar2，Ffar3，Ffar4，Fga，Fgb，Fgfr1，Fgg，Fgl1，Fgl2，Fgr，Flnb，Flot1，Fn1，Fndc4，Foxf1，Foxj1，Foxp1，Foxp3，Fpr1，Fpr2，Fpr3，Fpr-rs3，Fpr-rs4，Fpr-rs6，Fpr-rs7，Frk，Fut4，Fut7，Fyb，Fyb2，Fyn，Fzd5，G3bp1，G3bp2，Gab2，Gadd45g，Gal，Galp，Gapdh，Gapt，Garin5a，Gas6，Gata1，Gata2，Gata3，Gata6，Gba，Gbf1，Gbp2，Gbp2b，Gbp3，Gbp5，Gbp6，Gbp7，Gch1，Gcnt3，Gcsam，Gfer，Gfi1，Gfus，Ggt1，Ggt5，Ghrl，Ghsr，Gimap3，Gimap5，Git1，Gja1，Gm20547，Gm21970，Gm42543，Gm43302，Gm49352，Gp2，Gpatch3，Gper1，Gpld1，Gpr108，Gpr17，Gpr183，Gpr31b，Gpr33，Gpr4，Gprc5b，Gps2，Gpsm3，Gpx1，Gpx2，Gpx4，Gramd4，Grn，Grp，Gsdmd，Gsdme，Gsn，Gstp1，Gzmb，Gzmm，H2-Aa，H2-Ab1，H2bc1，H2-D1，H2-DMa，H2-DMb1，H2-DMb2，H2-Eb1，H2-Eb2，H2-K1，H2-L，H2-M1，H2-M10.1，H2-M10.2，H2-M10.3，H2-M10.4，H2-M10.5，H2-M10.6，H2-M2，H2-M3，H2-M5，H2-M9，H2-Oa，H2-Ob，H2-Q1，H2-Q10，H2-Q2，H2-Q4，H2-Q6，H2-Q7，H2-Q8，H2-Q9，H2-T22，H2-T23，H2-T3，H60b，H60c，Hamp，Hamp2，Havcr2，Hc，Hck，Hcst，Hdac5，Hdac7，Hdac9，Herc6，Hexim1，Hfe，Hgf，Hif1a，Hk1，Hlx，Hmces，Hmgb1，Hmgb2，Hmgb3，Hmgn2，Hmgn2-ps，Hmox1，Hnrnpa0，Hp，Hprt，Hps1，Hpse，Hpx，Hras，Hrg，Hrh4，Hsp90aa1，Hspa8，Hspd1，Hyal1，Hyal2，Hyal3，Icam1，Icosl，Ido1，Ier3，Ifi203，Ifi204，Ifi205，Ifi206，Ifi207，Ifi208，Ifi209，Ifi211，Ifi214，Ifi27，Ifi27l2b，Ifi35，Ifi44，Ifi44l，Ifih1，Ifit1，Ifit2，Ifit3，Ifitm1，Ifitm2，Ifitm3，Ifitm7，Ifna1，Ifna11，Ifna12，Ifna13，Ifna2，Ifna4，Ifna6，Ifna9，Ifnar1，Ifnar2，Ifnb1，Ifne，Ifng，Ifngr1，Ifngr2，Ifnk，Ifnl2，Ifnl3，Ifnlr1，Igf1，Igf1r，Igf2，Igha，Ighd，Ighg，Ighg1，Ighg2a，Ighg2b，Ighg2c，Ighg3，Ighm，Ighv1-61，Ighv1-62-3，Ighv1-72，Ighv3-6，Igkc，Igkv11-125，Igkv12-41，Igkv12-44，Igkv2-112，Igkv3-10，Igkv4-55，Igkv5-48，Igkv6-17，Igkv7-33，Igkv9-129，Iglc2，Iglc3，Igll1，Iglv2，Igtp，Iigp1，Ikbke，Ikbkg，Il10，Il12a，Il12b，Il12rb1，Il12rb2，Il13，Il13ra2，Il15，Il16，Il17a，Il17b，Il17c，Il17d，Il17f，Il17ra，Il17rb，Il17rc，Il17re，Il18，Il18bp，Il18r1，Il18rap，Il1a，Il1b，Il1f10，Il1r1，Il1rap，Il1rl1，Il1rl2，Il1rn，Il2，Il21，Il21r，Il22，Il22ra2，Il23a，Il23r，Il25，Il27，Il27ra，Il2ra，Il3，Il31ra，Il33，Il34，Il36a，Il36b，Il36g，Il36rn，Il4，Il4i1，Il4i1b，Il4ra，Il5，Il5ra，Il6，Il6ra，Il7，Il7r，Il9，Il9r，Ilrun，Inava，Inhca，Inpp5d，Inppl1，Ipo7，Irak1，Irak1bp1，Irak2，Irak3，Irak4，Irf1，Irf3，Irf4，Irf5，Irf7，Irf8，Irgm1，Irgm2，Isg15，Isg20，Isl1，Itch，Itfg2，Itga2，Itgal，Itgam，Itgav，Itgb1，Itgb2，Itgb2l，Itgb6，Itgb8，Itih4，Itk，Itm2a，Ivl，Jag1，Jak2，Jak3，Jam3，Jchain，Jun，Kars，Kat5，Kcnn4，Kdelr1，Kdm5d，Kdm6b，Khdrbs1，Kif16b，Kif5b，Kit，Kl，Klhl6，Klk1b1，Klk5，Klk7，Klkb1，Klrb1，Klrb1a，Klrb1b，Klrb1c，Klrb1f，Klrc1，Klrc2，Klrc3，Klrd1，Klre1，Klrg1，Klri1，Klri2，Klrk1，Kmt5b，Kmt5c，Kng1，Kng2，Krt1，Krt16，Krt6a，Kynu，Lacc1，Lag3，Lamp1，Lamp3，Laptm5，Lat，Lat2，Lax1，Lbp，Lck，Lcn2，Lcp1，Lcp2，Ldlr，Leap2，Lef1，Lep，Letmd1，Lfng，Lgals1，Lgals2，Lgals3，Lgals4，Lgals6，Lgals8，Lgals9，Lgr4，Lias，Lif，Lig4，Lilra5，Lilrb4a，Lilrb4b，Lime1，Lipa，Loxl3，Lpcat3，Lpin1，Lpl，Lpxn，Lrfn5，Lrp1，Lrp8，Lrrc14，Lrrc19，Lrrfip2，Lrrk2，Lsm14a，Lst1，Lta，Ltb，Ltb4r1，Ltb4r2，Ltbr，Ltf，Lxn，Ly86，Ly9，Ly96，Lyar，Lyn，Lypd10，Lyst，Macir，Mad2l2，Malt1，Map2k3，Map3k14，Map3k5，Map3k7，Map4k2，Mapk1，Mapk8，Mapkapk2，Mapkapk3，Marchf1，Marchf2，Marchf8，Marco，Mas1，Masp1，Masp2，Matr3，Mavs，Mbl1，Mbl2，Mcoln1，Mcoln2，Mcph1，Mdk，Mecom，Med1，Mef2c，Mefv，Mep1b，Metrnl，Mettl3，Mfap4，Mfhas1，Mfng，Mfsd6，Mgll，Mid2，Mif，Mill1，Mill2，Milr1，Mkrn2，Mlh1，Mmp12，Mmp7，Mmp8，Mndal，Mog，Morc3，Mpeg1，Mptx1，Mr1，Mrc1，Mrgpra1，Mrgpra2a，Mrgpra3，Mrgpra4，Mrgpra5，Mrgpra6，Mrgpra7，Mrgpra8，Mrgpra9，Mrgprb1，Mrgprb2，Mrgprb3，Mrgprb4，Mrgprb5，Mrgprb8，Mrgprx2，Ms4a1，Ms4a2，Msh2，Msh6，Msmp，Msrb1，Mst1r，Mtor，Muc19，Muc4，Mul1，Mvk，Myb，Myd88，Mylk3，Myo18a，Myo1c，Myo1f，Myo1g，Myo5a，N4bp1，Naip1，Naip2，Naip5，Naip6，Naip7，Nampt，Napepld，Nbn，Ncf1，Nckap1l，Ndfip1，Ndst1，Ndufc2，Ndufs4，Nectin2，Nedd4，Nepn，Nfam1，Nfatc2，Nfe2l1，Nfe2l2，Nfil3，Nfkb1，Nfkb2，Nfkbia，Nfkbib，Nfkbid，Nfkbil1，Nfkbiz，Ninj1，Nkg7，Nkiras2，Nkx2-3，Nlrc3，Nlrc4，Nlrc5，Nlrp10，Nlrp12，Nlrp14，Nlrp1a，Nlrp1b，Nlrp2，Nlrp3，Nlrp4a，Nlrp4b，Nlrp4c，Nlrp4e，Nlrp4f，Nlrp5，Nlrp6，Nlrp9a，Nlrp9b，Nlrp9c，Nlrx1，Nmb，Nmbr，Nmi，Nod1，Nod2，Nono，Nop53，Nos2，Notch1，Notch2，Nploc4，Nppa，Nppb，Nppc，Npy，Npy5r，Nr1d1，Nr1d2，Nr1h3，Nr1h4，Nr4a3，Nras，Nrros，Nsd2，Nt5e，Nts，Nupr1，Oas1a，Oas1c，Oas1d，Oas1e，Oas1f，Oas1g，Oas1h，Oas2，Oas3，Oasl1，Oasl2，Odam，Olfm4，Olr1，Oprk1，Optn，Orai1，Orm1，Orm2，Oscar，Osm，Otop1，Otub1，Otud4，Otud5，Otud7b，Otulin，P01629，P01630，P01631，P01634，P01635，P01636，P01637，P01638，P01641，P01643，P01644，P01645，P01646，P01647，P01648，P01649，P01652，P01653，P01654，P01655，P01656，P01657，P01658，P01659，P01660，P01661，P01662，P01663，P01664，P01665，P01666，P01667，P01668，P01669，P01670，P01671，P01672，P01673，P01674，P01675，P01676，P01677，P01678，P01679，P01680，P01723，P01724，P01725，P01726，P01727，P01728，P01729，P01741，P01745，P01746，P01747，P01748，P01750，P01753，P01756，P01757，P01759，P01786，P01787，P01788，P01789，P01790，P01791，P01792，P01793，P01794，P01795，P01796，P01797，P01798，P01799，P01800，P01801，P01802，P01803，P01804，P01807，P01809，P01810，P01811，P01812，P01819，P01820，P01822，P01823，P01843，P01851，P01852，P01864，P01878，P01881，P01882，P01896，P01915，P03976，P03977，P03980，P03987，P04224，P04940，P04941，P04943，P04944，P04946，P06329，P06330，P06344，P18524，P18525，P18526，P18527，P18528，P18529，P18530，P18532，P20040，P2rx1，P2rx7，P2ry14，P84750，Padi4，Pag1，Pagr1a，Panx1，Park7，Parp14，Parp3，Parp4，Parp9，Pawr，Paxip1，Pbk，Pbxip1，Pcbp2，Pck1，Pdcd1，Pdcd1lg2，Pdcd4，Pde12，Pde2a，Pde4b，Pde4d，Pde5a，Pdpk1，Peli1，Peli3，Pf4，Pgc，Pglyrp1，Pglyrp2，Pglyrp3，Pglyrp4，Phb，Phb2，Phf11a，Phpt1，Pi4k2a，Pianp，Pigr，Pik3ap1，Pik3cd，Pik3cg，Pik3r6，Pirb，Pja2，Pkn1，Pla2g10，Pla2g1b，Pla2g2d，Pla2g2e，Pla2g2f，Pla2g3，Pla2g4a，Pla2g5，Pla2g6，Pla2g7，Plaa，Plcg1，Plcg2，Plcl2，Pld2，Pld3，Pld4，Plec，Plekha1，Plekhm2，Plgrkt，Plp1，Plpp6，Plscr1，Plscr2，Pml，Pmp22，Pms2，Pnma1，Pnp，Pnp2，Polb，Polr3a，Polr3b，Polr3c，Polr3d，Polr3e，Polr3f，Polr3g，Polr3h，Polr3k，Pomc，Pou2af1，Pou2f2，Ppara，Ppard，Pparg，Ppbp，Ppl，Ppp1r14b，Ppp2r3c，Ppp3cb，Ppp6c，Pqbp1，Pram1，Prcp，Prdm1，Prdx1，Prdx2，Prf1，Prg2，Prg3，Prg4，Prkca，Prkcb，Prkcd，Prkce，Prkch，Prkcq，Prkcz，Prkd1，Prkd2，Prkdc，Prnp，Proc，Prr7，Psen1，Psen2，Psg17，Psg22，Psma1，Psmb4，Pspc1，Pstpip1，Ptafr，Ptgdr，Ptgds，Ptger1，Ptger2，Ptger3，Ptger4，Ptges，Ptgfr，Ptgir，Ptgis，Ptgs1，Ptgs2，Ptk2，Ptk2b，Ptk6，Ptn，Ptpn2，Ptpn22，Ptpn6，Ptprc，Ptprd，Ptprj，Ptprs，Ptx3，Pum1，Pum2，Pvr，Pvrig，Pxk，Pycard，Rab11fip2，Rab11fip5，Rab12，Rab17，Rab20，Rab27a，Rab29，Rab2b，Rab43，Rab44，Rab7b，Rabgef1，Rac2，Raet1a，Raet1b，Raet1c，Raet1d，Raet1e，Rag1，Rag2，Rap1a，Rap1gds1，Rapgef1，Rara，Rarres2，Rasgrp1，Rb1，Rbck1，Rbm14，Rbpj，Rc3h1，Rc3h2，Reg1，Reg2，Reg3a，Reg3b，Reg3d，Reg3g，Rel，Rela，Relb，Rftn1，Rgcc，Rhbdd3，Rictor，Rif1，Riok3，Ripk1，Ripk2，Ripk3，Rnase2a，Rnase2b，Rnase6，Rnf125，Rnf135，Rnf166，Rnf168，Rnf185，Rnf187，Rnf19b，Rnf31，Rnf8，Romo1，Rora，Rorc，Rpl13a，Rpl30，Rpl39，Rps19，Rps3，Rps6，Rps6ka3，Rps6ka4，Rps6ka5，Rps6kb1，Rsad2，Rtn4，S100a14，S100a7a，S100a8，S100a9，S1pr3，Saa1，Saa2，Saa3，Saa4，Sac3d1，Samhd1，Samsn1，Sarm1，Sbno2，Scap，Scgb1a1，Scimp，Scn9a，Scnn1b，Scyl1，Scyl3，Sdc1，Sdhaf4，Sec14l1，Sectm1a，Sectm1b，Seh1l，Sele，Selenos，Selp，Sema4a，Sema7a，Senp7，Serinc3，Serinc5，Serpina1b，Serpina3g，Serpina3n，Serpinb1a，Serpinb9，Serpinb9b，Serpinb9c，Serpinb9d，Serpinb9e，Serpinb9f，Serpinb9g，Serpinb9h，Serpine1，Serpinf1，Serpinf2，Serping1，Setd2，Setd4，Sfpq，Sftpd，Sgms1，Sh2b2，Sh2d1a，Sh2d1b1，Sh2d1b2，Sharpin，Shb，Shfl，Shld1，Shld2，Shld3，Shmt2，Shpk，Sigirr，Siglece，Siglecg，Sin3a，Sirpa，Sirt1，Sit1，Skap1，Skint1，Skint10，Skint11，Skint2，Skint3，Skint4，Skint5，Skint6，Skint7，Skint8，Skint9，Skp2，Sla，Sla2，Slamf1，Slamf6，Slamf7，Slamf8，Slamf9，Slc11a1，Slc15a2，Slc15a3，Slc15a4，Slc22a13，Slc26a6，Slc39a10，Slc46a2，Slc7a2，Slit2，Slpi，Smad1，Smad3，Smad6，Smad7，Smcr8，Smo，Smpd1，Smpdl3b，Snap23，Snca，Snx4，Socs1，Socs3，Socs5，Sod1，Sp100，Sp110，Spag11b，Spata2，Sphk1，Sphk2，Spi1，Spink5，Spink7，Spn，Spns2，Spon2，Sppl2a，Sppl2b，Sppl3，Sprr2a1，Src，Srebf1，Srms，Ssc5d，St3gal1，Stab1，Stap1，Stard7，Stat1，Stat2，Stat3，Stat5a，Stat5b，Stat6，Sting1，Stk11，Stk39，Stoml2，Stx11，Stx4a，Stx7，Stx8，Stxbp1，Stxbp2，Stxbp3，Stxbp4，Styk1，Sucnr1，Supt6，Susd4，Swap70，Syk，Syncrip，Syt11，Tac1，Tac4，Tafa3，Tap1，Tap2，Tapbp，Tapbpl，Tarm1，Tasl，Tbc1d23，Tbk1，Tbkbp1，Tbx21，Tbxa2r，Tcim，Tcirg1，Tec，Tespa1，Tfe3，Tfeb，Tff2，Tfrc，Tgfb1，Tgfb2，Tgfbr3，Tgtp1，Thbs1，Themis，Themis2，Thoc1，Thy1，Ticam1，Ticam2，Tifa，Timp1，Tirap，Tkfc，Tlr1，Tlr11，Tlr12，Tlr13，Tlr2，Tlr3，Tlr4，Tlr5，Tlr6，Tlr7，Tlr8，Tlr9，Tmem106a，Tmem33，Tmem43，Tmem98，Tnc，Tnf，Tnfaip3，Tnfaip6，Tnfaip8l2，Tnfrsf11a，Tnfrsf13b，Tnfrsf13c，Tnfrsf14，Tnfrsf17，Tnfrsf1a，Tnfrsf1b，Tnfrsf21，Tnfrsf4，Tnfsf10，Tnfsf11，Tnfsf12，Tnfsf13，Tnfsf13b，Tnfsf14，Tnfsf15，Tnfsf18，Tnfsf4，Tnfsf8，Tnfsf9，Tnip1，Tnip2，Tnip3，Tnk1，Tnk2，Tollip，Tomm70a，Tpsb2，Tradd，Traf2，Traf3，Traf3ip2，Traf4，Traf6，Trafd1，Trat1，Trem1，Trem2，Trem3，Treml4，Trex1，Trf，Trgv1，Tril，Trim10，Trim11，Trim12a，Trim12c，Trim13，Trim14，Trim15，Trim17，Trim21，Trim25，Trim26，Trim27，Trim28，Trim29，Trim30a，Trim30d，Trim31，Trim32，Trim34a，Trim35，Trim38，Trim39，Trim40，Trim41，Trim43a，Trim43b，Trim43c，Trim5，Trim55，Trim56，Trim58，Trim59，Trim6，Trim60，Trim62，Trim65，Trim68，Trim75，Trim8，Triml1，Triml2，Trp53，Trp53bp1，Trp73，Trpm4，Trpv1，Trpv4，Tsc1，Tslp，Tspan2，Tspan6，Ttbk1，Ttc4，Ttll1，Ttll12，Twist1，Twist2，Txk，Tyk2，Tyro3，Tyrobp，Uaca，Ubash3a，Ubd，Ube2k，Ube2n，Ubqln1，Ufd1，Ulbp1，Ulk4，Umod，Unc13d，Unc93b1，Ung，Usp12，Usp14，Usp15，Usp25，Usp27x，Usp29，Usp46，Usp9x，Vamp2，Vamp3，Vamp4，Vamp7，Vamp8，Vav1，Vav3，Vegfa，Vim，Vip，Vnn1，Vpreb1，Vps26b，Vps35，Vsig4，Vsir，Vtcn1，Vtn，Wap，Was，Washc1，Wdfy1，Wdr41，Wdr83，Wfdc1，Wfdc11，Wfdc12，Wfdc13，Wfdc15a，Wfdc15b，Wfdc17，Wfdc18，Wfdc2，Wfdc21，Wfdc3，Wfdc9，Wnk1，Wnk4，Wnt5a，Wrnip1，Xbp1，Xcl1，Xcr1，Xrcc4，Xrcc5，Xrcc6，Yes1，Ythdf2，Ythdf3，Ywhaz，Zap70，Zbp1，Zbtb1，Zbtb7b，Zc3h12a，Zc3hav1，Zcchc3，Zdhhc1，Zdhhc11，Zdhhc5，Zfp35，Zfp36，Zfp580，Zfp683，Zfp809，Znfx1，Zp3，Zp3r，Zyx | A2M, ABCC1, ABCF1, ABHD12, ABL1, ACE, ACE2, ACER3, ACKR1, ACKR2, ACKR3, ACKR4, ACOD1, ACP5, ACTG1, ACTR2, ACTR3, ACVR1, ADA, ADAM15, ADAM17, ADAM8, ADAMDEC1, ADAMTS12, ADAMTS13, ADAR, ADARB1, ADCY1, ADCY7, ADCY8, ADGRB1, ADGRE1, ADGRE2, ADGRE5, ADIPOQ, ADM, ADORA1, ADORA2A, ADORA2B, ADORA3, ADRA2A, AFAP1L2, AGER, AGT, AGTR1, AGTR2, AHCY, AHR, AHSG, AICDA, AIF1, AIM2, AIMP1, AIRE, AKAP1, AKAP8, AKIRIN2, AKNA, AKT1, ALCAM, ALOX15, ALOX5, ALOX5AP, ALPK1, AMBP, ANG, ANKHD1, ANKRD17, ANO6, ANXA1, ANXA3, AOAH, AOC3, AP1G1, AP3B1, APBB1IP, APCS, APLF, APLN, APOA1, APOA2, APOA4, APOBEC3A, APOBEC3B, APOBEC3C, APOBEC3D, APOBEC3F, APOBEC3G, APOBEC3H, APOE, APOL1, APOL2, APOL3, APP, APPL1, APPL2, AQP4, AREL1, ARG1, ARG2, ARHGEF2, ARID5A, ARL8B, ARRB2, ASH1L, ASS1, ATAD3A, ATAD5, ATG12, ATG5, ATM, ATP1B1, ATP6V0A2, ATP7A, ATRN, AXL, AZGP1, AZI2, AZU1, B2M, B4GALT1, BACH2, BAG6, BANF1, BANK1, BAP1, BATF, BAX, BCAR1, BCL10, BCL2, BCL3, BCL6, BCL6B, BCR, BDKRB1, BDKRB2, BIRC2, BIRC3, BLK, BLNK, BMI1, BMP2, BMP6, BMPR1A, BMPR1B, BMX, BPGM, BPI, BPIFA1, BPIFB1, BPIFB3, BRAF, BRD4, BST1, BST2, BTK, BTLA, BTN1A1, BTN2A1, BTN2A2, BTN2A3P, BTN3A1, BTN3A2, BTN3A3, BTNL10, BTNL2, BTNL3, BTNL8, BTNL9, C12orf4, C17orf99, C1QA, C1QB, C1QBP, C1QC, C1QTNF12, C1QTNF3, C1R, C1RL, C1S, C2, C2CD4A, C2CD4B, C3, C3AR1, C4A, C4B, C4BPA, C4BPB, C5, C5AR1, C5AR2, C6, C7, C8A, C8B, C8G, C9, C9JQL5, CACNB3, CACNB4, CACTIN, CADM1, CALCA, CALCOCO2, CALCRL, CAMK1D, CAMK2N1, CAMK4, CAMP, CARD11, CARD18, CARD8, CARD9, CASP1, CASP12, CASP4, CASP5, CASP6, CASP8, CAV1, CBL, CBLB, CCDC92, CCL1, CCL11, CCL13, CCL14, CCL15, CCL16, CCL17, CCL18, CCL19, CCL2, CCL20, CCL21, CCL22, CCL23, CCL24, CCL25, CCL26, CCL27, CCL28, CCL3, CCL3L1, CCL4, CCL4L1, CCL5, CCL7, CCL8, CCN3, CCN4, CCR1, CCR10, CCR2, CCR3, CCR4, CCR5, CCR6, CCR7, CCR8, CCR9, CCRL2, CD14, CD160, CD163, CD164, CD177, CD180, CD19, CD1A, CD1B, CD1C, CD1D, CD1E, CD2, CD200, CD200R1, CD200R1L, CD209, CD22, CD226, CD24, CD244, CD247, CD27, CD274, CD276, CD28, CD300A, CD300LF, CD33, CD36, CD38, CD3D, CD3E, CD3G, CD4, CD40, CD40LG, CD44, CD46, CD47, CD48, CD55, CD58, CD59, CD5L, CD6, CD68, CD7, CD70, CD74, CD79A, CD79B, CD80, CD81, CD83, CD84, CD86, CD8A, CD8B, CD8B2, CD96, CDC37, CDC42, CDC42EP2, CDC42EP4, CDH17, CDH5, CDK19, CDO1, CEACAM1, CEACAM8, CEBPA, CEBPB, CEBPG, CELA1, CELF1, CERS6, CERT1, CFB, CFD, CFH, CFHR1, CFHR2, CFHR3, CFHR4, CFHR5, CFI, CFP, CGAS, CH25H, CHGA, CHI3L1, CHIA, CHID1, CHIT1, CHST1, CHST2, CHST4, CHUK, CIITA, CITED1, CLC, CLCF1, CLDN1, CLEC10A, CLEC12B, CLEC2A, CLEC4A, CLEC4C, CLEC4D, CLEC4E, CLEC4G, CLEC4M, CLEC5A, CLEC6A, CLEC7A, CLNK, CLOCK, CLPB, CLU, CMA1, CMKLR1, CMTM3, CNIH1, CNOT7, CNPY3, CNR1, CNR2, CNTF, COCH, COL3A1, COLEC10, COLEC11, COLEC12, CORO1A, CPLX2, CR1, CR1L, CR2, CRACR2A, CRCP, CREB3L3, CREBBP, CRH, CRHBP, CRHR1, CRIP1, CRISP3, CRK, CRKL, CRP, CRTAM, CSF1, CSF1R, CSF2, CSF2RB, CSF3, CSK, CSMD3, CSRP3, CST7, CST9, CST9L, CST9LP1, CTLA4, CTNNBIP1, CTSC, CTSG, CTSH, CTSK, CTSL, CTSS, CTSV, CTSW, CUL3, CX3CL1, CX3CR1, CXCL1, CXCL10, CXCL11, CXCL12, CXCL13, CXCL14, CXCL16, CXCL17, CXCL2, CXCL3, CXCL5, CXCL6, CXCL8, CXCL9, CXCR1, CXCR2, CXCR3, CXCR4, CXCR5, CXCR6, CYBA, CYBB, CYBC1, CYLD, CYP11B1, CYP19A1, CYP26B1, CYP27B1, CYRIB, CYSLTR1, CYSLTR2, DAB2IP, DAGLA, DAGLB, DAPK1, DAPK3, DBNL, DCLRE1C, DCST1, DDT, DDX1, DDX21, DDX3X, DDX58, DDX60, DEFA1, DEFA3, DEFA4, DEFA5, DEFA6, DEFB1, DEFB104A, DEFB105A, DEFB106A, DEFB107A, DEFB108A, DEFB108B, DEFB110, DEFB112, DEFB113, DEFB114, DEFB116, DEFB118, DEFB119, DEFB121, DEFB123, DEFB124, DEFB125, DEFB126, DEFB127, DEFB128, DEFB131A, DEFB131B, DEFB132, DEFB133, DEFB134, DEFB135, DEFB136, DEFB4A, DENND1B, DGKZ, DHRS7B, DHX15, DHX36, DHX58, DHX9, DLL1, DMBT1, DNASE1, DNASE1L3, DNASE2, DOCK10, DOCK11, DOCK2, DPP8, DRD2, DROSHA, DTX3L, DUOXA1, DUOXA2, DUSP10, DUSP22, DUSP3, DYSF, EBI3, ECM1, ECSIT, EDA, EDN1, EDNRB, EIF2AK1, EIF2AK2, EIF2AK4, EIF2B1, EIF2B2, EIF2B3, EIF2B4, EIF2B5, ELANE, ELF1, ELF3, ELF4, EMP2, ENDOD1, ENDOU, ENPP1, ENPP2, ENPP3, ENTPD7, EOMES, EP300, EPG5, EPHA2, EPHB2, EPO, EPRS1, ERAP1, ERAP2, ERBIN, ERCC1, EREG, ERMAP, ESR1, ETS1, EXO1, EXOSC3, EXOSC6, EXOSC9, EXT1, EZR, F11R, F12, F2, F2R, F2RL1, F3, F6UB75, F8, FABP4, FADD, FAM3A, FANCA, FANCD2, FAS, FASLG, FASN, FAU, FBXO38, FBXO9, FCAMR, FCAR, FCER1A, FCER1G, FCER2, FCGR1A, FCGR1BP, FCGR2A, FCGR2B, FCGR2C, FCGR3A, FCGR3B, FCGRT, FCHO1, FCN1, FCN2, FCN3, FCRL3, FCRL4, FCRLB, FEM1A, FER, FER1L5, FES, FFAR2, FFAR3, FFAR4, FGA, FGB, FGL1, FGL2, FGR, FKBP1A, FLNB, FLOT1, FN1, FNDC4, FOLR2, FOS, FOXF1, FOXJ1, FOXP1, FOXP3, FPR1, FPR2, FPR3, FRK, FTH1, FURIN, FUT4, FUT7, FYB1, FYB2, FYN, FZD5, G3BP1, G3BP2, GAB2, GAL, GALP, GAPDH, GAPT, GARIN5A, GATA1, GATA2, GATA3, GATA6, GBA, GBF1, GBP1, GBP2, GBP3, GBP4, GBP5, GBP6, GBP7, GCH1, GCSAM, GCSAML, GEM, GFER, GFI1, GGT1, GGT2P, GGT3P, GGT5, GHRL, GHSR, GIT1, GNL1, GNLY, GP2, GPATCH3, GPER1, GPI, GPLD1, GPR108, GPR151, GPR17, GPR183, GPR31, GPR32, GPR32P1, GPR33, GPR4, GPR65, GPR68, GPRC5B, GPS2, GPSM3, GPX1, GPX4, GRAMD4, GRN, GRP, GSDMD, GSN, GSTP1, GTPBP1, GZMA, GZMB, GZMM, H0Y3Z8, H0Y858, H2BC1, H2BC10, H2BC11, H2BC12, H2BC12L, H2BC21, H7C3V1, HAMP, HAVCR2, HCK, HCST, HDAC4, HDAC5, HDAC9, HERC5, HEXIM1, HFE, HGF, HHLA2, HIF1A, HK1, HLA-A, HLA-B, HLA-C, HLA-DMA, HLA-DMB, HLA-DOA, HLA-DOB, HLA-DPA1, HLA-DPB1, HLA-DQA1, HLA-DQA2, HLA-DQB1, HLA-DQB2, HLA-DRA, HLA-DRB1, HLA-DRB3, HLA-DRB4, HLA-DRB5, HLA-E, HLA-F, HLA-G, HLA-H, HLX, HMCES, HMGB1, HMGB2, HMGB3, HMGN2, HMHB1, HMOX1, HMSD, HNRNPA0, HP, HPR, HPRT1, HPX, HRAS, HRG, HRH1, HRH2, HRH4, HSP90AA1, HSPA1A, HSPA1B, HSPD1, HSPG2, HYAL1, HYAL2, HYAL3, ICAM1, ICOS, ICOSLG, IDO1, IFI16, IFI27, IFI35, IFI44, IFI44L, IFI6, IFIH1, IFIT1, IFIT1B, IFIT2, IFIT3, IFIT5, IFITM1, IFITM2, IFITM3, IFNA1, IFNA10, IFNA14, IFNA16, IFNA17, IFNA2, IFNA21, IFNA4, IFNA5, IFNA6, IFNA7, IFNA8, IFNAR1, IFNAR2, IFNB1, IFNE, IFNG, IFNGR1, IFNGR2, IFNK, IFNL1, IFNL2, IFNL3, IFNL4, IFNLR1, IFNW1, IGF1, IGF1R, IGFBP4, IGHA1, IGHA2, IGHD, IGHD1-1, IGHE, IGHG1, IGHG2, IGHG3, IGHG4, IGHJ1, IGHM, IGHV1-18, IGHV1-2, IGHV1-24, IGHV1-3, IGHV1-38-4, IGHV1-45, IGHV1-46, IGHV1-58, IGHV1-69, IGHV1-69-2, IGHV1-69D, IGHV1-8, IGHV1OR15-1, IGHV1OR15-9, IGHV1OR21-1, IGHV2-26, IGHV2-5, IGHV2-70, IGHV2-70D, IGHV2OR16-5, IGHV3-11, IGHV3-13, IGHV3-15, IGHV3-16, IGHV3-20, IGHV3-21, IGHV3-23, IGHV3-30, IGHV3-30-3, IGHV3-30-5, IGHV3-33, IGHV3-35, IGHV3-38, IGHV3-38-3, IGHV3-43, IGHV3-43D, IGHV3-48, IGHV3-49, IGHV3-53, IGHV3-64, IGHV3-64D, IGHV3-66, IGHV3-7, IGHV3-72, IGHV3-73, IGHV3-74, IGHV3-9, IGHV3OR15-7, IGHV3OR16-10, IGHV3OR16-12, IGHV3OR16-13, IGHV3OR16-17, IGHV3OR16-8, IGHV4-28, IGHV4-30-2, IGHV4-30-4, IGHV4-31, IGHV4-34, IGHV4-38-2, IGHV4-39, IGHV4-4, IGHV4-59, IGHV4-61, IGHV4OR15-8, IGHV5-10-1, IGHV5-51, IGHV6-1, IGHV7-4-1, IGHV7-81, IGHV8-51-1, IGKC, IGKJ1, IGKV1-12, IGKV1-13, IGKV1-16, IGKV1-17, IGKV1-27, IGKV1-33, IGKV1-37, IGKV1-39, IGKV1-5, IGKV1-6, IGKV1-8, IGKV1-9, IGKV1D-12, IGKV1D-13, IGKV1D-16, IGKV1D-17, IGKV1D-33, IGKV1D-37, IGKV1D-39, IGKV1D-42, IGKV1D-43, IGKV1D-8, IGKV1OR2-108, IGKV2-24, IGKV2-28, IGKV2-29, IGKV2-30, IGKV2-40, IGKV2D-24, IGKV2D-26, IGKV2D-28, IGKV2D-29, IGKV2D-30, IGKV2D-40, IGKV3-11, IGKV3-15, IGKV3-20, IGKV3-7, IGKV3D-11, IGKV3D-15, IGKV3D-20, IGKV3D-7, IGKV3OR2-268, IGKV4-1, IGKV5-2, IGKV6-21, IGKV6D-21, IGKV6D-41, IGLC1, IGLC2, IGLC3, IGLC6, IGLC7, IGLJ1, IGLL1, IGLL5, IGLV10-54, IGLV11-55, IGLV1-36, IGLV1-40, IGLV1-44, IGLV1-47, IGLV1-50, IGLV1-51, IGLV2-11, IGLV2-14, IGLV2-18, IGLV2-23, IGLV2-33, IGLV2-8, IGLV3-1, IGLV3-10, IGLV3-12, IGLV3-16, IGLV3-19, IGLV3-21, IGLV3-22, IGLV3-25, IGLV3-27, IGLV3-32, IGLV3-9, IGLV4-3, IGLV4-60, IGLV4-69, IGLV5-37, IGLV5-39, IGLV5-45, IGLV5-48, IGLV5-52, IGLV6-57, IGLV7-43, IGLV7-46, IGLV8-61, IGLV9-49, IGSF6, IKBKB, IKBKE, IKBKG, IL10, IL10RB, IL12A, IL12B, IL12RB1, IL13, IL13RA2, IL15, IL16, IL17A, IL17B, IL17C, IL17D, IL17F, IL17RA, IL17RC, IL17RE, IL18, IL18BP, IL18R1, IL18RAP, IL19, IL1A, IL1B, IL1F10, IL1R1, IL1R2, IL1RAP, IL1RL1, IL1RL2, IL1RN, IL2, IL20, IL20RB, IL21, IL21R, IL22, IL22RA2, IL23A, IL23R, IL25, IL27, IL27RA, IL2RA, IL2RG, IL3, IL31RA, IL32, IL33, IL34, IL36A, IL36B, IL36G, IL36RN, IL37, IL4, IL4I1, IL4R, IL5, IL5RA, IL6, IL6R, IL6ST, IL7, IL7R, IL9, IL9R, ILRUN, INAVA, INPP5D, INPPL1, INS, IPO7, IRAK1, IRAK1BP1, IRAK2, IRAK3, IRAK4, IRF1, IRF3, IRF4, IRF5, IRF7, IRF8, IRGM, ISG15, ISG20, ISL1, ITCH, ITFG2, ITGA2, ITGAD, ITGAL, ITGAM, ITGB1, ITGB2, ITGB6, ITGB8, ITIH4, ITK, ITM2A, JAG1, JAK1, JAK2, JAK3, JAM3, JCHAIN, JUN, JUNB, KAAG1, KARS1, KAT5, KCNN4, KDM5D, KDM6B, KHDRBS1, KIF5B, KIR2DL1, KIR2DL3, KIR2DL4, KIR2DS1, KIR2DS2, KIR2DS5, KIR3DL1, KIR3DS1, KIT, KL, KLF4, KLHL6, KLK3, KLK5, KLK7, KLKB1, KLRB1, KLRC1, KLRC2, KLRC3, KLRC4, KLRD1, KLRF2, KLRG1, KLRK1, KMT5B, KMT5C, KNG1, KRT1, KRT16, KRT6A, KYNU, LACC1, LAG3, LAIR1, LAMP1, LAMP3, LAPTM5, LAT, LAT2, LAX1, LBP, LCK, LCN2, LCP1, LCP2, LDLR, LEAP2, LEF1, LEP, LETMD1, LFNG, LGALS1, LGALS2, LGALS3, LGALS4, LGALS9, LGR4, LIAS, LIF, LIG4, LILRA1, LILRA2, LILRA3, LILRA4, LILRA5, LILRA6, LILRB1, LILRB2, LILRB3, LILRB4, LILRB5, LIME1, LIMK1, LIPA, LOC102723996, LOXL3, LPCAT3, LPL, LPXN, LRFN5, LRP1, LRP8, LRRC14, LRRC19, LRRK2, LSM14A, LST1, LTA, LTB, LTB4R, LTB4R2, LTBR, LTF, LXN, LY75, LY86, LY9, LY96, LYAR, LYN, LYST, LYZ, MACIR, MAD2L2, MADCAM1, MALT1, MAP2K3, MAP2K4, MAP2K6, MAP2K7, MAP3K1, MAP3K14, MAP3K5, MAP3K7, MAP4K2, MAPK1, MAPK10, MAPK13, MAPK14, MAPK7, MAPK8, MAPK9, MAPKAPK2, MAPKAPK3, MAPT, MARCHF1, MARCHF2, MARCHF8, MARCO, MAS1, MASP1, MASP2, MATR3, MAVS, MBL2, MBP, MCOLN1, MCOLN2, MCPH1, MDK, MED1, MEF2C, MEFV, MEP1B, METRNL, METTL3, MFAP4, MFHAS1, MFNG, MGLL, MGST2, MICA, MICB, MID2, MIF, MILR1, MKRN2, MLH1, MMP12, MMP25, MMP26, MMP3, MMP8, MMP9, MNDA, MOG, MORC3, MPEG1, MR1, MRC1, MRGPRX1, MRGPRX2, MS4A1, MS4A2, MSH2, MSH6, MSMP, MSRB1, MST1R, MTOR, MT-RNR2, MUC7, MUL1, MVK, MX1, MX2, MYB, MYD88, MYL11, MYLK3, MYO1C, MYO1G, N4BP1, N4BP3, NAIP, NAPEPLD, NBN, NCF1, NCF2, NCKAP1L, NCR1, NCR3, NCR3LG1, NDFIP1, NDST1, NDUFC2, NECTIN1, NECTIN2, NFAM1, NFATC2, NFATC3, NFATC4, NFE2L2, NFIL3, NFILZ, NFKB1, NFKB2, NFKBIA, NFKBIB, NFKBID, NFKBIL1, NFKBIZ, NFX1, NINJ1, NKG7, NKIRAS2, NKX2-3, NLRC3, NLRC4, NLRC5, NLRP1, NLRP10, NLRP11, NLRP12, NLRP13, NLRP14, NLRP2, NLRP2B, NLRP3, NLRP4, NLRP5, NLRP6, NLRP7, NLRP8, NLRP9, NLRX1, NMB, NMBR, NMI, NOD1, NOD2, NONO, NOP53, NOS2, NOTCH1, NOTCH2, NOX1, NOX4, NPLOC4, NPY, NPY5R, NR1D1, NR1D2, NR1H2, NR1H3, NR1H4, NR4A3, NRROS, NSD2, NT5E, NUB1, NUPR1, OAS1, OAS2, OAS3, OASL, ODAM, OGG1, OLR1, OPRD1, OPRK1, OPRM1, OPTN, ORAI1, ORM1, ORM2, OSM, OSMR, OTOP1, OTUB1, OTUD4, OTUD7B, OTULIN, P2RX1, P2RX7, PADI4, PAG1, PAK1, PAK2, PAK3, PARK7, PARP1, PARP14, PARP3, PARP4, PARP9, PAWR, PAXIP1, PBK, PBXIP1, PCBP2, PCK1, PDCD1, PDCD1LG2, PDCD4, PDE12, PDE2A, PDE4B, PDE4D, PDPK1, PELI1, PF4, PF4V1, PGC, PGLYRP1, PGLYRP2, PGLYRP3, PGLYRP4, PHB1, PHB2, PHF14, PHPT1, PI3, PIANP, PIGR, PIK3AP1, PIK3CA, PIK3CD, PIK3CG, PIK3R1, PIK3R2, PIK3R3, PIK3R5, PIK3R6, PJA2, PKHD1L1, PKN1, PLA2G10, PLA2G1B, PLA2G2A, PLA2G2D, PLA2G2E, PLA2G2F, PLA2G3, PLA2G4A, PLA2G4B, PLA2G4C, PLA2G5, PLA2G6, PLA2G7, PLAA, PLCG1, PLCG2, PLCL2, PLD2, PLD3, PLD4, PLEC, PLEKHA1, PLEKHM2, PLGRKT, PLK2, PLP1, PLPP4, PLPP6, PLSCR1, PML, PNMA1, PNP, POLB, POLR3A, POLR3B, POLR3C, POLR3D, POLR3E, POLR3F, POLR3G, POLR3H, POLR3K, POU2AF1, POU2F2, PPARA, PPARD, PPARG, PPBP, PPP1R14B, PPP2R3C, PPP3CB, PPP6C, PQBP1, PRAM1, PRCP, PRDM1, PRDX1, PRDX2, PRDX5, PRF1, PRG2, PRG3, PRG4, PRKCB, PRKCD, PRKCE, PRKCH, PRKCQ, PRKCZ, PRKD1, PRKD2, PRKDC, PRKRA, PRNP, PROC, PROK2, PRR7, PRSS2, PRSS3, PRTN3, PSEN1, PSG9, PSMA1, PSMA6, PSMB10, PSMB4, PSPC1, PSTPIP1, PTAFR, PTGDR, PTGDR2, PTGDS, PTGER1, PTGER2, PTGER3, PTGER4, PTGES, PTGFR, PTGIR, PTGIS, PTGS1, PTGS2, PTK2, PTK2B, PTK6, PTN, PTPN1, PTPN11, PTPN2, PTPN22, PTPN6, PTPRC, PTPRD, PTPRJ, PTPRS, PTX3, PUM1, PUM2, PVR, PVRIG, PXDN, PXK, PYCARD, PYDC1, PYDC2, PYDC5, PYHIN1, RAB11FIP2, RAB17, RAB20, RAB27A, RAB29, RAB2B, RAB43, RAB44, RAB7B, RABGEF1, RAC1, RAC2, RAET1E, RAET1G, RAET1L, RAG1, RAG2, RAP1GAP, RAP1GAP2, RAPGEF3, RAPGEF4, RARA, RARRES2, RASGRP1, RB1, RBCK1, RBM14, RBPJ, RC3H1, RC3H2, REG1A, REG1B, REG3A, REG3G, REL, RELA, RELB, RFTN1, RFX1, RGCC, RGS1, RHBDD3, RHBDF2, RICTOR, RIF1, RIOK3, RIPK1, RIPK2, RIPK3, RNASE2, RNASE3, RNASE6, RNASE7, RNASE8, RNASET2, RNF125, RNF135, RNF166, RNF168, RNF185, RNF187, RNF19B, RNF31, RNF39, RNF8, ROMO1, RORA, RORC, RPL13A, RPL30, RPL39, RPS19, RPS3, RPS6, RPS6KA3, RPS6KA4, RPS6KA5, RPS6KB1, RSAD2, RTN4, S100A12, S100A13, S100A14, S100A7, S100A8, S100A9, S1PR3, S1PR4, SAA1, SAA2, SAA4, SAMHD1, SAMSN1, SANBR, SARM1, SASH3, SBNO2, SCAP, SCART1, SCG2, SCGB1A1, SCIMP, SCN9A, SCNN1B, SCUBE1, SCYL1, SCYL3, SDC1, SDHAF4, SEC14L1, SECTM1, SELE, SELENOS, SELP, SEMA3C, SEMA4A, SEMA4D, SEMA7A, SEMG1, SEMG2, SENP7, SERINC3, SERINC5, SERPINA1, SERPINA3, SERPINB4, SERPINB9, SERPINE1, SERPINF1, SERPINF2, SERPING1, SETD2, SETD4, SETD6, SFPQ, SFTPD, SH2B2, SH2D1A, SH2D1B, SHARPIN, SHB, SHFL, SHLD1, SHLD2, SHLD3, SHMT2, SHPK, SIGIRR, SIGLEC1, SIGLEC10, SIN3A, SIPA1, SIRPA, SIRT1, SIRT2, SIT1, SKAP1, SKP2, SLA, SLA2, SLAMF1, SLAMF6, SLAMF7, SLAMF8, SLAMF9, SLC11A1, SLC15A2, SLC15A3, SLC15A4, SLC22A13, SLC26A6, SLC30A8, SLC39A10, SLC46A2, SLPI, SMAD1, SMAD3, SMAD6, SMAD7, SMCR8, SMO, SMPD1, SMPDL3B, SNAP23, SNCA, SNX4, SOCS1, SOCS3, SOCS5, SOD1, SOS1, SP100, SP2, SPAG11A, SPAG11B, SPATA2, SPG21, SPHK1, SPHK2, SPI1, SPINK5, SPINK7, SPN, SPNS2, SPON2, SPP1, SPPL2A, SPPL2B, SPPL3, SPRR2A, SRC, SRMS, SRPK1, SRPK2, SSC5D, ST3GAL1, ST6GAL1, STAB1, STAP1, STAT1, STAT2, STAT3, STAT5B, STAT6, STING1, STK11, STK39, STMP1, STOML2, STX4, STX7, STX8, STXBP1, STXBP2, STXBP3, STXBP4, STYK1, SUCNR1, SUPT6H, SUSD4, SWAP70, SYK, SYNCRIP, SYT11, TAC1, TAC4, TACR1, TAFA3, TANK, TAP1, TAP2, TAPBP, TAPBPL, TARM1, TASL, TBK1, TBKBP1, TBX21, TBXA2R, TCF12, TCF7, TCIM, TCIRG1, TDGF1, TEC, TEK, TENM1, TESPA1, TF, TFE3, TFEB, TFR2, TFRC, TGFB1, TGFB2, TGFBR3, THBS1, THEMIS, THEMIS2, THOC1, THY1, TICAM1, TICAM2, TIFA, TIMP1, TIRAP, TKFC, TLR1, TLR10, TLR2, TLR3, TLR4, TLR5, TLR6, TLR7, TLR8, TLR9, TMED1, TMEM106A, TMEM33, TMEM43, TMEM98, TMIGD2, TMIGD3, TMSB4X, TNC, TNF, TNFAIP1, TNFAIP3, TNFAIP6, TNFAIP8L2, TNFRSF11A, TNFRSF13B, TNFRSF13C, TNFRSF14, TNFRSF17, TNFRSF1A, TNFRSF1B, TNFRSF21, TNFRSF4, TNFSF10, TNFSF11, TNFSF12, TNFSF13, TNFSF13B, TNFSF14, TNFSF15, TNFSF18, TNFSF4, TNFSF8, TNFSF9, TNIP1, TNIP2, TNIP3, TNK1, TNK2, TOLLIP, TOMM70, TP53, TP53BP1, TPST1, TRA, TRAC, TRADD, TRAF2, TRAF3, TRAF3IP2, TRAF4, TRAF6, TRAFD1, TRAJ3, TRAJ31, TRAJ42, TRAT1, TRAV10, TRAV1-1, TRAV1-2, TRAV12-1, TRAV12-2, TRAV12-3, TRAV13-1, TRAV13-2, TRAV14DV4, TRAV16, TRAV17, TRAV18, TRAV19, TRAV2, TRAV20, TRAV21, TRAV22, TRAV23DV6, TRAV24, TRAV25, TRAV26-1, TRAV26-2, TRAV27, TRAV29DV5, TRAV3, TRAV30, TRAV34, TRAV35, TRAV36DV7, TRAV38-1, TRAV38-2DV8, TRAV39, TRAV4, TRAV40, TRAV41, TRAV5, TRAV6, TRAV7, TRAV8-1, TRAV8-2, TRAV8-3, TRAV8-4, TRAV8-6, TRAV9-1, TRAV9-2, TRB, TRBC1, TRBC2, TRBD1, TRBJ1-1, TRBJ1-2, TRBJ1-3, TRBJ1-4, TRBJ1-5, TRBJ1-6, TRBJ2-1, TRBJ2-2, TRBJ2-3, TRBJ2-4, TRBJ2-5, TRBJ2-6, TRBJ2-7, TRBV10-1, TRBV10-2, TRBV10-3, TRBV11-1, TRBV11-2, TRBV11-3, TRBV12-3, TRBV12-4, TRBV12-5, TRBV13, TRBV14, TRBV16, TRBV17, TRBV18, TRBV19, TRBV2, TRBV20-1, TRBV23-1, TRBV24-1, TRBV25-1, TRBV27, TRBV28, TRBV29-1, TRBV30, TRBV3-1, TRBV4-1, TRBV4-2, TRBV4-3, TRBV5-1, TRBV5-3, TRBV5-4, TRBV5-5, TRBV5-6, TRBV5-7, TRBV5-8, TRBV6-1, TRBV6-2, TRBV6-3, TRBV6-4, TRBV6-5, TRBV6-6, TRBV6-7, TRBV6-8, TRBV6-9, TRBV7-1, TRBV7-2, TRBV7-3, TRBV7-4, TRBV7-6, TRBV7-7, TRBV7-8, TRBV7-9, TRBV9, TRDC, TRDD1, TRDJ1, TRDV1, TRDV2, TRDV3, TREM1, TREM2, TREML1, TREML4, TREX1, TRGC1, TRGC2, TRGJ1, TRGV1, TRGV10, TRGV11, TRGV2, TRGV3, TRGV4, TRGV5, TRGV8, TRGV9, TRIL, TRIM10, TRIM11, TRIM13, TRIM14, TRIM15, TRIM17, TRIM21, TRIM22, TRIM23, TRIM25, TRIM26, TRIM27, TRIM28, TRIM29, TRIM31, TRIM32, TRIM34, TRIM35, TRIM38, TRIM39, TRIM4, TRIM40, TRIM41, TRIM43, TRIM43B, TRIM48, TRIM49, TRIM49B, TRIM49C, TRIM49D1, TRIM5, TRIM51, TRIM51G, TRIM55, TRIM56, TRIM58, TRIM59, TRIM6, TRIM60, TRIM61, TRIM62, TRIM64, TRIM64B, TRIM64C, TRIM68, TRIM75, TRIM77, TRIM8, TRIML1, TRIML2, TRPM4, TRPV1, TRPV4, TSC1, TSLP, TSPAN2, TSPAN6, TTBK1, TTC4, TTLL1, TTLL12, TUBB, TUBB4B, TUSC2, TXK, TYK2, TYRO3, TYROBP, UBASH3A, UBD, UBE2K, UBE2N, UBQLN1, UCN, UFD1, UFL1, UGT1A1, ULBP1, ULBP2, ULBP3, UMOD, UNC13D, UNC93B1, UNG, USP14, USP15, USP17L2, USP18, USP27X, USP29, V9GYV3, VAMP2, VAMP3, VAMP7, VAMP8, VAV1, VAV2, VAV3, VCAM1, VIM, VNN1, VPREB1, VPREB3, VPS26B, VPS35, VSIG4, VSIR, VTCN1, VTN, WAS, WDFY1, WDR41, WDR83, WFDC1, WFDC10A, WFDC10B, WFDC11, WFDC12, WFDC13, WFDC2, WFDC3, WFDC5, WFDC9, WNK1, WNK4, WNT5A, WRNIP1, XBP1, XCL1, XCL2, XCR1, XIAP, XRCC5, XRCC6, YES1, YTHDF2, YTHDF3, ZAP70, ZBP1, ZBTB1, ZBTB7B, ZC3H12A, ZC3HAV1, ZCCHC3, ZDHHC1, ZDHHC11, ZDHHC18, ZDHHC5, ZFP36, ZFPM1, ZNF580, ZNF683, ZNFX1, ZP3, ZP4, ZYX |

**Table S2. Primers sequences used in the quantitative real-time PCR analysis**

| **Genes** | **Forward primer** | **Reverse primer** |
| --- | --- | --- |
| Gapdh | GGGTCCCAGCTTAGGTTCAT | CCAATACGGCCAAATCCGTTC |
| Saa1 | GGAGTCTGGGCTGCTGAGAAAA | TGTCTGTTGGCTTCCTGGTCAG |
| Icam1 | AAACCAGACCCTGGAACTGCAC | GCCTGGCATTTCAGAGTCTGCT |
| Fgl1 | GGAAACTGTGCTGAGGAAGAGC | TCCGTTTCTGCCCTGTAGGAAC |
| Cxcl14 | TACCCACACTGCGAGGAGAAGA | CGCTTCTCGTTCCAGGCATTGT |
| Ccl6 | CACCAGTGGTGGGTGCATCAAG | GTGCTTAGGCACCTCTGAACTC |
| S100a8 | CAAGGAAATCACCATGCCCTCTA | ACCATCGCAAGGAACTCCTCGA |
| S100a9 | TGGTGGAAGCACAGTTGGCAAC | CAGCATCATACACTCCTCAAAGC |
| S100a11 | GAAGGATGGAAACAACACTCAACT | CGTCACAGTTGAGGTCCAGCTT |
| Cxcl1 | TCCAGAGCTTGAAGGTGTTGCC | AACCAAGGGAGCTTCAGGGTCA |
| Il1b | TGGACCTTCCAGGATGAGGACA | GTTCATCTCGGAGCCTGTAGTG |
| Pias3 | CAAGAAGGCTCCCTATGAGTCG | GGTTTCATCGGACACCAGGATC |
| Nr0b2 | CCAAGGAGTATGCGTACCTGAAG | GCTCCAAGACTTCACACAGTGC |
| Stat3 | AGGAGTCTAACAACGGCAGCCT | GTGGTACACCTCAGTCTCGAAG |
| Socs3 | GGACCAAGAACCTACGCATCCA | CACCAGCTTGAGTACACAGTCG |
