# Supplementary material for: Stat3 activation-triggered transcriptional networks govern the early stage of HBV-induced hepatic inflammation
Source: mBio. 2024 Mar 5;15(4):e03068-23. doi: 10.1128/mbio.03068-23 (PMC11005361; doi:10.1128/mbio.03068-23)
Supplement: Supplemental text — List of reagents, animal models, human samples, instruments, and software. [file mbio.03068-23-s0003.doc]

- 1. **Antibodies**

| **Name** | **Citation** | **Supplier** | **Cat no.** | **Clone no.** |
| --- | --- | --- | --- | --- |
| Mouse anti-Stat3 |  | Abcam | ab119352 | 9D8 |
| Rabbit anti-Phospho-Stat3 |  | Abcam | ab76315 | EP2147Y |
| Violet660 anti-Mouse Cd11b |  | BioLegend | 101239 | M1/70 |
| PerCP/Cyanine5.5 anti-mouse Ly-6C |  | BioLegend | 128012 | HK1.4 |
| Brilliant Violet 421™ anti-mouse F4/80 |  | BioLegend | 123131 | BM8 |
| PE/Cyanine7 anti-mouse CD45 |  | BioLegend | 157206 | S18009F |
| Rabbit anti-NF-kB p65 |  | Cell Signaling | 8242S | D14E12 |
| Rabbit anti-NF-kB p65 (Phospho Ser536) |  | Cell Signaling | 3033T | 93H1 |
| β-Actin (13E5) Rabbit mAb |  | Cell Signaling | 4970S | 13E5 |
| Anti-rabbit IgG, HRP-linked Antibody |  | Cell Signaling | 7074P2 |  |
| Anti-mouse IgG, HRP-linked Antibody |  | Cell Signaling | 7076P2 |  |
| Monoclonal mouse anti-HBcAg |  | MXB Biotechnologies | MAB-0899 | MX104 |

- 1. **Cell lines**

| **Name** | **Citation** | **Supplier** | **Cat no.** | **Passage no.** | **Authentication test method** |
| --- | --- | --- | --- | --- | --- |
| **NA** |  |  |  |  |  |

- 1. **Organisms**

| **Name** | **Citation** | **Supplier** | **Strain** | **Sex** | **Age** | **Overall n number** |
| --- | --- | --- | --- | --- | --- | --- |
| Mice: HBV transgenic mice |  | Vitalstar Biotechnology (Beijing, China) | B6-Tg (HBV)5 Vst /Vst | Male | 1st month, 2nd month, 3rd month, 6th month | 93 |
| Mice: (WT) mice |  | Vitalstar Biotechnology (Beijing, China) | C57BL/6N | Male | 1st month, 2nd month, 3rd month, 6th month | 78 |

- 1. **Sequence based reagents**

| **Name** | **Sequence** | **Supplier** |
| --- | --- | --- |
| Forward primer of Gapdh | GGGTCCCAGCTTAGGTTCAT | Thermo |
| Reverse primer of Gapdh | CCAATACGGCCAAATCCGTTC | Thermo |
| Forward primer of Saa1 | GGAGTCTGGGCTGCTGAGAAAA | Thermo |
| Reverse primer of Saa1 | TGTCTGTTGGCTTCCTGGTCAG | Thermo |
| Forward primer of Icam1 | AAACCAGACCCTGGAACTGCAC | Thermo |
| Reverse primer of Icam1 | GCCTGGCATTTCAGAGTCTGCT | Thermo |
| Forward primer of Fgl1 | GGAAACTGTGCTGAGGAAGAGC | Thermo |
| Reverse primer of Fgl1 | TCCGTTTCTGCCCTGTAGGAAC | Thermo |
| Forward primer of Cxcl14 | TACCCACACTGCGAGGAGAAGA | Thermo |
| Reverse primer of Cxcl14 | CGCTTCTCGTTCCAGGCATTGT | Thermo |
| Forward primer of Ccl6 | CACCAGTGGTGGGTGCATCAAG | Thermo |
| Reverse primer of Ccl6 | GTGCTTAGGCACCTCTGAACTC | Thermo |
| Forward primer of S100a8 | CAAGGAAATCACCATGCCCTCTA | Thermo |
| Reverse primer of S100a8 | ACCATCGCAAGGAACTCCTCGA | Thermo |
| Forward primer of S100a9 | TGGTGGAAGCACAGTTGGCAAC | Thermo |
| Reverse primer of S100a9 | CAGCATCATACACTCCTCAAAGC | Thermo |
| Forward primer of S100a11 | GAAGGATGGAAACAACACTCAACT | Thermo |
| Reverse primer of S100a11 | CGTCACAGTTGAGGTCCAGCTT | Thermo |
| Forward primer of Cxcl1 | TCCAGAGCTTGAAGGTGTTGCC | Thermo |
| Reverse primer of Cxcl1 | AACCAAGGGAGCTTCAGGGTCA | Thermo |
| Forward primer of Il1b | TGGACCTTCCAGGATGAGGACA | Thermo |
| Reverse primer of Il1b | GTTCATCTCGGAGCCTGTAGTG | Thermo |
| Forward primer of Pias3 | CAAGAAGGCTCCCTATGAGTCG | Thermo |
| Reverse primer of Pias3 | GGTTTCATCGGACACCAGGATC | Thermo |
| Forward primer of Nr0b2 | CCAAGGAGTATGCGTACCTGAAG | Thermo |
| Reverse primer of Nr0b2 | GCTCCAAGACTTCACACAGTGC | Thermo |
| Forward primer of Stat3 | AGGAGTCTAACAACGGCAGCCT | Thermo |
| Reverse primer of Stat3 | GTGGTACACCTCAGTCTCGAAG | Thermo |
| Forward primer of Socs3 | GGACCAAGAACCTACGCATCCA | Thermo |
| Reverse primer of Socs3 | CACCAGCTTGAGTACACAGTCG | Thermo |

- 1. **Biological samples**

| **Description** | **Source** | **Identifier** |
| --- | --- | --- |
| Liver biopsies of chronic HBV infection cases | Ministry of Education Key Laboratory of Child Development and Disorders, Department of Infection, National Clinical Research Center for Child Health and Disorders, Children's Hospital of Chongqing Medical University, Chongqing, China. |  |

- 1. **Deposited data**

| **Name of repository** | **Identifier** | **Link** |
| --- | --- | --- |
| Gene Expression Omnibus (RNAseq data of mice) | In process | In process |

- 1. **Software**

| **Software name** | **Manufacturer** | **Version** |
| --- | --- | --- |
| R (windows) |  | 4.2.1 |
| Rstudio (windows) |  | 2022.02.1+461 |
| FlowJo |  | 10.4 |
| GraphPad Prism |  | 8.0 |
| CaseViewer |  | 2.4 |
| Image-Pro Plus |  | 6.0.0.260 |
| ImageJ |  | 1.52a |

- 1. **Other (*e.g*. drugs, proteins, vectors etc.)**

| **Name** | **Manufacturer** | **Identifier** |
| --- | --- | --- |
| WesternBright ECL | Advansta | K-12045-D50 |
| DMSO | Biofroxx | 67-68-5 |
| RBC lysis buffer | BioLegend | 420301 |
| Percoll | Cytiva | 17089101 |
| Protein ladder | Thermo | 26616 |
| TRIzol | Life technologies | 15596026 |
| Stattic | Selleck | S7024 |
| PrimeScriptTM RT reagent Kit with gDNA Eraser | Takara | RR047A |
| TB Green Premix Ex Taq II kit | Takara | RR820A |
| RNA isolation kit | ZYMO Research | R2052 |
| Primary antibody dilution buffer | Beyotime | P0023A |
| DAB Plus Kit | MXB Biotechnologies | DAB-2031 |
| Total proteins extraction kit | KeyGEN BioTECH | KGP250 |
| Bicinchoninic acid kit | KeyGEN BioTECH | KGPBCA |
| Hitachi 7600 Series automatic biochemical analyzer | Hitachi |  |
| COBAS® AmpliPrep/COBAS® TaqMan® HBV test kit (Version 2.0) | Roche Diagnostic |  |
| Chemiluminescence microparticle immunoassay (CMIA) with alinity HBsAg and HBeAg detection kits | Abbott Laboratories |  |
| Blocking reagent for IHC | ZSGB-BIO | ZLI-9021 |
| Digital section scanning analysis system | 3DHISTECH | Pannoramic DESK DW Ⅱ |
| Flow cytometer | Beckman Coulter | CytoFLEX |
| Enhanced chemiluminescence and gel imaging system | Bio-Rad | ChemicDoc^TM^ MP Imaging System |
| CFX Connect^TM^ Real-Time PCR System | Thermo |  |
| Microplate reader | Thermo |  |
